# Genetic models of cleavage-reduced and soluble TREM2 reveal distinct effects on myelination and microglia function in the cuprizone model

**DOI:** 10.1186/s12974-022-02671-z

**Published:** 2023-02-08

**Authors:** Nicolau Beckmann, Anna Neuhaus, Stefan Zurbruegg, Pia Volkmer, Claudia Patino, Stefanie Joller, Dominik Feuerbach, Arno Doelemeyer, Tatjana Schweizer, Stefan Rudin, Ulf Neumann, Ramon Berth, Wilfried Frieauff, Fabrizio Gasparini, Derya R. Shimshek

**Affiliations:** 1 Musculoskeletal Diseases Area, Novartis Institutes for BioMedical Research, Novartis Pharma AG, CH-4002 Basel, Switzerland; 2 Neuroscience, Novartis Institutes for BioMedical Research, Novartis Pharma AG, CH-4002 Basel, Switzerland; 3 Preclinical Safety, Novartis Institutes for BioMedical Research, Novartis Pharma AG, CH-4002 Basel, Switzerland

**Keywords:** TREM2, Microglia, Neuroinflammation, Phagocytosis, Cuprizone, MRI

## Abstract

Triggering receptor expressed on myeloid cells 2 (TREM2) is a cell-surface immunoreceptor expressed on microglia, osteoclasts, dendritic cells and macrophages. Heterozygous loss-of-function mutations in TREM2, including mutations enhancing shedding form the cell surface, have been associated with myelin/neuronal loss and neuroinflammation in neurodegenerative diseases, such as Alzheimer`s disease and Frontotemporal Dementia. Using the cuprizone model, we investigated the involvement of soluble and cleavage-reduced TREM2 on central myelination processes in cleavage-reduced (TREM2-IPD), soluble-only (TREM2-sol), knockout (TREM2-KO) and wild-type (WT) mice. The TREM2-sol mouse is a new model with selective elimination of plasma membrane TREM2 and a reduced expression of soluble TREM2. In the acute cuprizone model demyelination and remyelination events were reflected by a T2-weighted signal intensity change in magnetic resonance imaging (MRI), most prominently in the external capsule (EC). In contrast to WT and TREM2-IPD, TREM2-sol and TREM2-KO showed an additional increase in MRI signal during the recovery phase. Histological analyses of TREM2-IPD animals revealed no recovery of neuroinflammation as well as of the lysosomal marker LAMP-1 and displayed enhanced cytokine/chemokine levels in the brain. TREM2-sol and, to a much lesser extent, TREM2-KO, however, despite presenting reduced levels of some cytokines/chemokines, showed persistent microgliosis and astrocytosis during recovery, with both homeostatic (TMEM119) as well as activated (LAMP-1) microglia markers increased. This was accompanied, specifically in the EC, by no myelin recovery, with appearance of myelin debris and axonal pathology, while oligodendrocytes recovered. In the chronic model consisting of 12-week cuprizone administration followed by 3-week recovery TREM2-IPD displayed sustained microgliosis and enhanced remyelination in the recovery phase. Taken together, our data suggest that sustained microglia activation led to increased remyelination, whereas microglia without plasma membrane TREM2 and only soluble TREM2 had reduced phagocytic activity despite efficient lysosomal function, as observed in bone marrow-derived macrophages, leading to a dysfunctional phenotype with improper myelin debris removal, lack of remyelination and axonal pathology following cuprizone intoxication.

**Supplementary Information:**

The online version contains supplementary material available at 10.1186/s12974-022-02671-z.

## Introduction

Triggering receptor expressed on myeloid cells 2 (TREM2), a type I transmembrane glycoprotein expressed on microglia, dendritic cells, osteoclasts and other tissue-resident macrophages, can be shed from the cell surface and is a potential biomarker for Alzheimer`s disease (AD) in CSF [1–4]. In humans, TREM2 homozygous loss-of-function mutation carriers exhibit bone cysts and fractures during adolescence, together with an early onset frontal lobe syndrome and progressive presenile dementia known as Nasu-Hakola [5, 6]. Neuropathological findings include myelin and neuronal loss as well as increased neuroinflammation. Furthermore, strong genetic links of TREM2 mutations to AD, Frontotemporal Dementia (FTD), Parkinson`s disease (PD) and Amyotrophic Lateral Sclerosis (ALS) have been found [4]. It is hypothesized that TREM2 expression increases at the peak of disease and drives its resolution, by acting as a phagocytosis receptor, supporting microglia survival/migration and modulating cytokine release. In different disease contexts, it has been observed that absence of functional TREM2 seems to lock microglia in a homeostatic and/or not fully activated phenotype and thus, to slow down autophagy and hamper resolution of inflammation [7–11]. On the other side, it has been shown that antibodies that enhance TREM2 activity induce a protective microglia phenotype [12, 13]. In addition, cleavage-reduced TREM2 mice show an acceleration of microglia response in the context of Aβ pathology [14].

The cuprizone-induced demyelination model enables to study different myelination and neuroinflammation stages in the central nervous system [15–20]. TREM2 knockout (TREM2-KO) mice displayed altered microglia responses to cuprizone intoxication as well as reduced clearance of myelin debris [21, 22]. Furthermore, it has been observed that TREM2 is essential for remyelination processes in the lysolecithin model [23]. In addition, TREM2 contributes to cleaning up toxic lipids in multiple sclerosis [24]. Soluble TREM2 can be observed in body fluids, mainly by shedding from the cell surface, serving as a potential biomarker of microglia activation in neurodegenerative diseases [25]. Furthermore, a shedding enhancing TREM2 variant (H157Y) has been associated with AD risk [26]. However, the role of soluble TREM2 in disease context is up-to-now unclear.

Here, we investigated the involvement of sustained TREM2 activation and soluble TREM2 on myelination and neuroinflammation processes in the cuprizone model using genetically modified animals and applying magnetic resonance imaging (MRI) and histology readouts [27, 28]. We show that sustained TREM2 activation in the cleavage-reduced TREM2-IPD mouse increases microglia activation and enhances remyelination in the recovery phase, while soluble-only TREM2 (TREM2-sol) leads to dysfunctional microglia with distinct differences to microglia lacking TREM2 (TREM2-KO), in particular proper lysosomal function and increase in the lysosomal marker LAMP-1. On the other hand, both TREM2-sol and TREM2-KO lead to enhanced myelin debris, axonal pathology, and absence of remyelination in the recovery phase.

## Materials and methods

### Statement on animal welfare

In vivo experimental procedures followed the Swiss animal welfare regulations. Studies described in this report were approved by the Swiss Cantonal Veterinary Authority of Basel City, Switzerland, and performed under the license number BS-2711. Authors complied with the ARRIVE guidelines for animal experimentation.

### Animals

TREM2-KO mice [29] were purchased from the UC Davis KOMP Repository (Project ID VG10093) and were then bred at Novartis Pharma AG (Basel, Switzerland) in the C57BL/6 background. Wild-type (WT) mice were either littermates from a TREM2 heterozygous KO breeding or C57BL/6J from Charles River (Sulzfeld, Germany). TREM2-IPD mice were generated as described earlier [14]. TREM2-sol (TREM2 soluble-only, B6-Trem2em3Npa) knock-in (KI) mice were generated at the Novartis Institutes for BioMedical Research (NIBR, Cambridge, USA). In brief, two single guide RNAs (sgRNAs) and a donor oligo (200 bp) containing the stop-codon TGA after the amino acid histidine 157 and including a restricting enzyme site Eco57I (CACTGAAGC) replacing the wild-type amino-acids HST (CACAGCACC) were designed to target the mouse TREM2 allele. The sgRNAs (sgRNA1, complementary sequence: 5’-gggaccactactgtacct; sgRNA2, forward sequence: 5’-aagtggaacacagcacctcc) were ordered as crisp RNAs (crRNAs) from Integrated DNA Technologies (IDT, Coralville, IA, USA) as part of their Alt-R CRISPR/Cas9 system. sgRNA1 or sgRNA2 was microinjected, along with the universal Alt-R tracrRNA (IDT), donor oligo and Cas9 protein (PNA Bio, Thousand Oaks, CA, USA), into the pro-nucleus of fertilized C57Bl/6J oocytes. Viable two-cell embryos were re-implanted in pseudo-pregnant B6CBAF1 females. The resulting pups were genotyped by PCR analysis (primer F1: 5`-agctacccgctactgcaaag, primer R1: 5`-cccgatgagctcttccacat, expected product length: 635 bp, PCR program: 95 °C 3 min, 95 °C 20 s, 60 °C 20 s, 72 °C 45 s, go to step 2, 35 cycles, 72 °C 5 min, 14 °C forever), followed by XhoI digestion and Sanger sequencing. Eight KI founders were back-crossed with C57BL/6J mice (JAX stock #000664, The Jackson Laboratory, Bar Harbor, ME, USA) and the F0 and F1 generation was subjected to MiSeq sequencing (Illumina Inc, San Diego, CA, USA) using a forward primer 5′-atgctggagatctctgggtcc and a reverse primer 5′-gtgagttgctacaaagggctcc to generate amplicons, and NuGEN’s Amplicon Sequencing System (part number 9092-256, Tecan, Redwood City, CA, USA) for the construction of a Next-Generation Sequencing library. Two founder lines were chosen. One mouse of each founder line (heterozygous) was subjected to targeted locus amplification (TLA) at Cergentis (Utrecht, The Netherlands). For this, viable frozen mouse spleen cells were used and processed according to Cergentis’ TLA protocol [30]. Two primer sets were designed on the transgene (Set 1: reverse primer exon 3: 5′-GGTCATCTAGAGGGTCTGTA, forward primer exon 3: 5′-TTCGTGCACTTAGTAGATCC; Set 2: reverse primer HA: 5′-CTACCTGGGTTTGTCCATG, forward primer HA: 5′-GAACGTTAGCCTGTCTCTAG). The primer sets were used in individual TLA amplifications. PCR products were purified, library prepared using the Illumina Nextera flex protocol and sequenced on an Illumina sequencer. Reads were mapped using the Burrows–Wheeler Aligner's Smith–Waterman (BWA–SW) alignment algorithm [31], version 0.7.15-r1140, settings bwasw -b 7. The NGS reads were aligned to the transgene sequence and the host genome. The mouse mm10 genome was used as host reference genome sequence. The presence of single-nucleotide variants (SNVs) was determined using samtools mpileup (samtools version 1.3.1, Genome Research Limited, Hinxton, UK) [32, 33]. Fusion sequences consisting of two parts of the transgene were identified using a proprietary Cergentis script. Fusions resulting from the TLA procedure itself were recognized by the restriction enzyme-specific sequence at the junction site and removed. Integration sites were detected based on: (a) coverage peak(s) in the genome, and (b) the identification of fusion-reads between the transgene sequence and the host genome. The transgenes of both samples were integrated in mouse chr17:48,351,169-48,351,193 as intended. Genotyping has been performed from ear punch or tail tip DNA by two different PCRs with GotTaq (Promega, Madison, WI, USA) and the following primers: (i) PCR with Knock-in primer sets 996_sTrem2-KI_F (5′-gatagggaatcgaccagaggc) and 997_sTrem2-KI_R (5′-ctactgtacctactcgtgcttcag) as well as internal control for beta-actin (747-beta-actin-fw-1: 5′-TGTGGGCATTTGATGAGCCGG; 748-beta-actin-rev-1: 5′-AAGACCCAGAGGCCATTGAGG); (ii) PCR with wild-type primer sets 994_sTrem2-wt_F (5′-tggaacacagcacctccagg) and 995_sTrem2-wt_R (5′-gggcttcatgtggctcaggg) as well as internal control for beta-actin with primer pair (747-beta-actin-fw-1 and 748-beta-actin-rev-1). Expected KI band size: 251 bp, wild-type band size: 468 bp. Internal beta-actin control: 122 bp. PCR program: 95 °C 3 min, 95 °C 30 s, 60 °C 30 s, 72 °C 30 s, go to step 2, 35 cycles, 72 °C 5 min, 8 °C forever.

Except were specified, all animals were female, 2–3 months of age at the beginning of the study, and divided into *n* = 4–7 mice per group. For the RNA analyses wild-type, TREM2-sol and TREM2-KO mice were 12 months old. Genotyping of TREM2-KO mice was performed according to the protocol by UC Davis KOMP. All animals were allowed to adapt for 7 days prior to the start of the experiment and housed in individual ventilated cages (max. 4 mice/XJ Type cage). Mice were given access to food and water ad libitum throughout the study.

### Cuprizone treatment

Animals were treated with cuprizone for 5 (acute model) or 12 (chronic model) weeks and then switched to normal food for 3 or 4 weeks. Cuprizone [Bis(cyclohexanone) oxaldihydrazone, Sigma-Aldrich, Buchs, Switzerland] was mixed into rodent food pellets (0.2% w/w) by Granovit AG (Kaiseraugst, Switzerland). Two studies for the acute model were performed (studies ran at different timepoints). In one study: (i) homozygous TREM2-KO and wild-type (named wt2) were included while in the other (ii) homozygous TREM2-IPD, homozygous TREM2-sol and wild-type (named wt1) were used. For both studies the wild-type data is shown separately to account for possible differences in the cuprizone-induced pathology. In vivo and post-mortem analyses were performed blind to the genotype and treatment of the mice.

### Magnetic resonance imaging (MRI)

Measurements were performed with a Biospec 70/30 spectrometer (Bruker Medical Systems, Ettlingen, Germany) operating at 7T. The operational software of the scanner was Paravision 5.1 (Bruker). Images were acquired from anesthetized, spontaneously breathing animals using a circularly polarized coil (Bruker, Model 1P T20063V3; internal diameter 23 mm) for radiofrequency excitation and detection. Neither cardiac nor respiratory triggering was applied. Following a short period of introduction in a box, mice were maintained in anesthesia with 1.5% isoflurane (Abbott, Cham, Switzerland) in oxygen, administered via a nose cone. During MRI signal acquisitions, animals were placed in prone position in a cradle made of Plexiglas, the body temperature was kept at 37 ± 1 °C using a heating pad, and the respiration was monitored.

A T_2_-weighted, two-dimensional multislice RARE (Rapid Acquisition with Relaxation Enhancement) sequence [34] was used for selecting the regions-of-interest (ROIs) and for evaluating signal intensities. A two-dimensional multislice gradient-recalled FLASH (Fast Low-Angle Shot) acquisition [35] served to assess the magnetization transfer ratio (MTR), a measure reflecting myelin content (Beckmann et al., 2018). Assessments of the relaxation time T_2_ were performed using a multislice spin-echo sequence. As the three sequences had the same anatomical parameters, the ROIs for evaluations were selected on the RARE images and then transferred to the FLASH and spin-echo images. MRI images were analyzed using the Paravision software.

The parameters of the acquisitions were the following: (a) RARE sequence: effective echo time (TE) 80 ms, repetition time (TR) 3280 ms, RARE factor 16, 12 averages. Hermite pulses of duration/bandwidth 1 ms/5400 Hz and 0.64 ms/5344 Hz were used for radiofrequency excitation and refocusing, respectively. Fat suppression was achieved by a gauss512 pulse of 2.61 ms/1051 Hz duration/bandwidth followed by a 2-ms-long gradient spoiler. The total acquisition time was of 7 min 52.3 s; (b) FLASH sequence: TE/TR 2.8/252.8 ms, 4 averages. A hermite pulse of 0.9 ms/6000 Hz duration/bandwidth and flipangle 30° was used for radiofrequency excitation. MTR contrast was introduced by a gauss pulse of 15 ms/182.7 Hz duration/bandwidth applied with radiofrequency peak amplitude of 7.5 μT and an irradiation offset of 2500 Hz. The acquisition was then repeated with the same parameters but without the introduction of the MTR contrast. MTR was then computed using the formula MTR = (S_0_ − S_MTR_)/S_0_, where S_0_ and S_MTR_ represent, respectively, the signal intensities in the FLASH acquisitions without and with the introduction of the MTR contrast. The total acquisition time for both data sets was of 6 min 31.6 s; (c) assessments of the relaxation time T_2_ were performed using a multislice spin-echo sequence with the parameters: 16 echoes spaced by 11 ms, TE from 11 to 176 ms, TR 3000 ms, fat suppression as described above. T_2_ was determined by exponentially fitting with Origin Pro^®^ 2021 (OriginLab, Northampton, MA, USA) the mean signals as function of TE from ROIs placed in the corpus callosum (cc) and the external capsule (ec). All three sequences were run using the same anatomical parameters: field-of view 20 × 18 mm^2^, matrix size 213 × 192, pixel size 0.094 × 0.094 mm^2^, slice thickness 0.5 mm, 15 adjacent slices.

### Post-mortem analyses

Prior to cull mice were perfused trans-cardiacally by phosphate-buffered saline under isoflurane anesthesia and blood was withdrawn by heart-puncture. Brains were removed from the skull and the forebrain (excluding olfactory bulb, brainstem and cerebellum) was used for biochemical assessments and histology. In the study with the TREM2-KO animals were also perfused with 4% paraformaldehyde. All brains subjected for histology were fixed in 4% paraformaldehyde for 48 h at 4 °C.

### Mouse TREM2 determination from brain and cell culture supernatant

Mouse forebrain was homogenized in 1:10 (w/v) Tris-buffered saline (TBS) containing Complete protease inhibitor (Roche Diagnostics, Rotkreuz, Switzerland) using an ultrasonic device. 50 μl of TBS containing Complete Protease inhibitor were mixed with 50 µl of homogenate and incubated on ice, with regular vortexing for 15 min. The extract was centrifuged for 15 min at 100,000×*g* and the clear supernatant (50 µl) was transferred to the ELISA plate. Medium from cultures of bone marrow derived macrophages (BMDM) was collected, spun down at 1000 RPM for 5 min and 25 µl of the supernatant was added to the coated 96-well plates. Polyclonal sheep anti-TREM2 antibody AF1729 (R&D Systems, Minneapolis, MN, USA) at 0.2 mg/ml in PBS was used as coating antibody. Samples were incubated for 2 h and the plate was washed. For detection, wells were filled with 50 µl of 0.5 µg/ml of biotinylated anti-TREM2 antibody AF1729 (R&D Systems), incubated for 1 h at room temperature and washed. Streptavidine–horseradish peroxidase (# 893975, R&D Systems) and tetramethylbenzidine/H_2_O_2_ substrate were added at room temperature, and the reaction was stopped after 20 min by addition of 2 N sulfuric acid. Absorbance at 450 nm was read with a SpectraMax Plus 384 reader (Molecular Devices, San Jose, CA, USA). Recombinant mouse TREM2 (Novoprotein, Wuijang, China, Cat # CM92) from 39 to 2500 pg/ml was used as standard.

### Cytokine/chemokine measurements

Supernatant from BMDM was taken on in vitro day 7. Half brains were dounce homogenized 1:10 (w:v) in TBS, aliquoted and stored at − 80 °C. A radioimmunoprecipitation assay buffer (Merck Millipore 20-188) was added ten times to a brain homogenate aliquot, mixed and incubated on ice for 10 min, then centrifuged for 5 min at 10,000 rcf at 4 °C. Supernatants from brain and from BMDM (1:2 or 1:10 in diluent 41) were used for U-plex (mouse) Meso Scale Discovery (Acro Biosystems, Newark, DE, USA) electrochemiluminescence analyses according to a manufacturer’s protocol.

### Mouse plasma neurofilament-light (NF-L) measurements

Plates (MesoScale Discovery (MSD), Rockville, MD, USA; Multi Array 96 well, L15XA-3) were coated with 25 µl capture antibody working solution 25 µl/well (Uman Diagnostics, Umeå, Sweden; α-NF-L mAb 47:3 (UD1); 2.35 mg/ml; diluted in PBS to 0.5 ug/ml), shaked for 10 min and incubated overnight at 4 °C. Plates were washed 3× with 150 µl wash buffer [0.05% Tween20 (Sigma, CAS 9005-64-5) in 1xTBS (Sigma, T5912-1L)]. Blocking was performed with 150 µl blocking buffer/well (2% BSA (fraction V) in wash buffer) and incubated during 1 h at room temperature. Plates were again washed 3× with 150 µl wash buffer. Standard (NF-L, Uman Diagnostics, 27001) and plasma samples [dilution on plate 1:2 in 1xTBS with complete [protease inhibitor cocktail tablets (Roche, Basel, Switzerland)] were added [25 µl per well, in sample dilution buffer (0.1% BSA in wash buffer)] and plates were sealed and incubated for 2 h at room temperature. Plates were washed 3× with 150 µl wash buffer. Detection antibody (Uman Diagnostics, biotin labelled α-NF-L mAb 2:1 (UD3); 0.86 mg/ml; diluted to 0.5 μg/ml in 0.5% BSA;) was added [25 µl per well in detection antibody dilution buffer (0.5% BSA in wash buffer)] and plates were sealed and incubated for 1 h at room temperature. Plates were washed 3× with 150 µl wash buffer. Streptavidin SULFO TAG (MSD SulfoTag, CAT R32AD-1, 500 µg/ml working dilution: 1:1000 in PBS) was added (25 µl/well) and incubated for 30–60 min at room temperature in the dark. Plates were washed 3× with 150 µl wash buffer. 2× read buffer (Cat. R92TC-3, working solution 1:2 in destilled water) was added (150 µl/well) and plates were analyzed with the plate reader (MSD SECTOR Imager 600).

### RNA isolation and qRT-PCR

Total RNA was isolated from the forebrain of wild-type, TREM2-sol and TREM2-KO mice using TRIzol™ reagent (Invitrogen, Waltham, MA, USA) and the RNA concentrations were measured using a NanoDrop 2000 spectrophotometer (Fisher Scientific, Waltham, MA). The isolated RNA (1 μg) was treated with DNaseI (Invitrogen) and reverse transcribed into cDNA using SuperScript III First-Strand Synthesis SuperMix for qRT-PCR (Invitrogen). To evaluate the expression levels of the target gene and endogenous reference we used FastStart Universal SYBR Green Master {ROX} (Roche) with the ABI StepOnePlus Real-Time PCR system (ThermoFisher Scientific, Waltham, MA, USA). The sequences of forward and reverse primers for mTREM2 were spanning exon 2 and 3 (fw: 5′-CACCATCACTCTGAAGAACC-3′; rev: 5′-GGAGTCATCGAGTTTCGAGG-3′) and GAPDH (fw: 5′-AACTCCCACTCTTCCACCTT-3′; rev: 5′-GAGTAAGAAACCCTGGAGGA-3′) (Microsynth). The values were normalized to those of GAPDH using equation: 2^−ΔΔCt^ = 2^−^^(ΔCttarget – Avg ΔCtControl)^.

### Primary microglia isolation from mouse pup brains

Eight mouse pup brains (*n* = 2 brains from each genotype: WT, TREM2-KO, TREM2-IPD, TREM2-sol) were used for the glial cell isolation and processed as described earlier [14]. The isolated cells were re-suspended in microglia medium containing DMEM supplemented with GlutaMAX (Gibco, 10569010), 10% Fetal Bovine Serum (FBS) (Gibco, 10082147), 1% Pen/Strep (Gibco, 15070063), 1% Sodium Pyruvate (Gibco, 11360070) and 1% nonessential amino acids (NEAA) (Gibco, 11140035). Mixed glial cultures were incubated in Poly-l-Lysine (Sigma-Aldrich, P4707) pre-coated T75 culture flasks containing 10 ml of microglia medium at 37 °C in 5% CO_2_. After 13–20 days in vitro, the culture flasks were placed on an orbital shaker for 60 min to detach the microglia from the astrocyte layer. The microglia-containing medium was collected, centrifuged at 300 x *g* for 4 min at room temperature, and the cell count was determined.

### Isolation, culturing and differentiation of bone-marrow-derived macrophages (BMDM)

BMDM were prepared from adult WT, TREM2-KO, TREM2-IPD, and TREM2-sol mice (*n* = 2 from each genotype) as described previously [14]. Cells were re-suspended in RPMI1640 culture media (Gibco, 61870010) containing 10% FBS, 1% Pen/Strep, 1% Sodium pyruvate, 1% NEAA, 0.025 M HEPES (Gibco, 15630080), 50 mM 2-Mercaptoethanol (Gibco, 31350010) and seeded in 10 cm culture dishes with 10 ml of medium. Murine macrophage colony-stimulating factor (M-CSF) (R&D Systems, 416-ML-050) with a final concentration of 40 ng/ml was added to the medium, and the BMDM were incubated for 4 days at 37 °C in 5% CO_2_. On day 5 of incubation new culture medium containing 40 ng/ml M-CSF plus either 20 ng/ml murine interleukin 4 (IL-4) (R&D Systems, 404-ML-050) and 40 ng/ml murine interleukin 13 (IL-13) (R&D Systems, 413-ML-005) or 50 ng/ml murine Interferon-gamma (IFN-γ) (BioConcept, Allschwil, Switzerland, 315-05-100UG) was added to the cells. The cells were then incubated for at least 48 h at 37 °C in 5% CO_2_. For harvesting, the BMDM were detached with Tryp LE Express (Gibco, 12605010).

### Labeling of primary myelin with pHrodo Green

Three hemispheres from PBS-perfused brains of two WT animals were put in one gentleMACS C-tube (Miltenyi Biotech, 130-093-237) and processed separately. According to the adult brain dissociation kit (Miltenyi Biotech, 130-107-677) the brain tissue was dissociated and the suspension was filtered through a 70 µm cell strainer and filled up to 10 ml with PBS. After centrifugation at 1500 rpm for 10 min at room temperature two times, the pellet was re-suspended in 3100 µl PBS, and a 900 µl debris removal solution was added. The solution was gently overlaid with cold PBS and centrifuged at 3000 x *g* for 10 min at 4 °C. The myelin layer was collected and washed with PBS. 500 μl of the myelin suspension were centrifuged at 3000 x *g* for 10 min at 4 °C and then re-suspended in a 0.1 M sodium bicarbonate buffer at 20 mg/ml. The pHrodo Green STP Ester (Thermo Fisher, P35369) was added at a final concentration of 100 µM to the myelin suspension. After 45 min of incubation, the myelin suspension was centrifuged at 13000 x *g* for 10 min at room temperature and three times washed with PBS. Finally, the labeled myelin was re-suspended in sterile PBS and stored at 4 °C. Unlabeled myelin was collected and stored at 4 °C.

### Phagocytosis assay

Microglia were plated at a seeding density of 20,000 cells and BMDM at a seeding density of 30,000 cells per well in a 96-well black/clear flat bottom microplate (Thermo Scientific Nunc 96-well plate) with 100 µl medium per well. pHrodo-myelin and pHrodo Green S. aureus (Invitrogen, Waltham, MA, USA, P35367) were diluted in microglia/BMDM medium and added to the cells for up to 24 h. The plates were analyzed with an IncuCyte S3 Live-cell Analysis System (Sartorius, Goettingen, Germany). The integrated fluorescence intensities were analyzed by the IncuCyte Software, and the area under the curve (AUC) was calculated by GraphPad Prism (Dotmatics, San Diego, CA, USA).

### Fluorescence-activated cell sorting (FACS) analysis of cell surface and intracellular TREM2

Cells were re-seeded in 6-well plates and treated with 5 µM DPC333 [36] (DPC) overnight or 50 ng/ml Phorbol-12-myristate-13-acetate (PMA) (Sigma Aldrich, 16561-29-8) for 30 min, and then detached using Accutase (StemCell Technologies, Koeln, Germany) and transferred to a falcon tube (Corning, Corning, NY, USA). Afterwards, cells were treated with an anti-mouse CD16/CD32 antibody (eBioscience, ThermoFisher, 14016185) in FACS buffer (PBS containing 2% FBS, 0.5 mM EDTA, and 0.05% Sodium azide) for 20 min at 4 °C. Next, the cells were transferred to a 96-well clear V-Bottom microplate and stained with a human/mouse TREM2 Alexa Fluor 488 antibody (R&D Systems FAB17291G) or a rat IgG2A Alexa Fluor^®^ 488-conjugated isotype control (R&D Sytems, IC006G), for 30 min at 4 °C. Staining was detected with a BD FACS Canto II Flow Cytometer (BD Biosciences, Franklin Lakes, USA) and analyzed using FlowJo (BD Biosciences). The gate for TREM2 positive cells was set based on the isotype control (threshold 0.2% positive cells in the isotype control). For intracellular TREM2 cells were permeabilized with permeabilization buffer (BD Biosciences, 554722) for 20 min at 4 °C.

### Cathepsin B assay for analyzing the endo-lysosomal activity

Differentiated BMDM were plated in a 96-well black/clear flat bottom microplate at a cell density of 15,000 cells in 100 µl medium per well. After 24 h of incubation, cells were treated with 100 µl of DMSO (0.1%) (Sigma-Aldrich, D8418), Bafilomycin A1 (100 nM) (Sigma-Aldrich, 19-148), or K-18 (3 µM) (Novartis Pharma AG, Basel, Switzerland) and incubated for 24 h at 37 °C in 5% CO_2_. On the next day, Magic Red substrate from the Magic red Cathepsin B Kit (Bio-Rad, ICT938) was diluted in distilled water (dH_2_O) (Invitrogen, 10977055) containing Hoechst (1:4000) according to the manufacturer’s protocol, and 3.8 µl of the dilution were added to each well. Cells were incubated at 37 °C in the dark for 45 min and washed with PBS twice. The In-Cell Analyzer 2500 HS (Cytiva, Marlborough, MA, USA) was used to detect fluorescence signals, which were quantified using ImageJ/Fiji (version 1.2; WS Rasband, National Institute of Health, Bethesda, MD) and normalized to the nuclei count.

### ATP-based cell survival assay

After differentiation, BMDM were re-seeded in a 96-well flat-bottom microplate at a cell density of 30,000 cells in 100 µl medium (with/without M-CSF) per well and incubated for 2 and 3.5 days, respectively, at 37 °C in 5% CO_2_. After 48 h/84 h, 100 µl of CellTiter-Glo (Promega, G9242) was added to each well, and the plates were incubated for 15 min at room temperature in the dark. Luminescence signals were measured with a 2104 EnVision Multilabel Plate Reader (PerkinElmer, Waltham, MA, USA).

### Histology of brains

After fixation, brains were processed for paraffin embedding by dehydration through increasing ethanol series. Paraffin sections of 3 μm thickness (coronal brain sections from the preoptic area, coordinates according to Paxino and Franklin: interaural 4.9–3.94 mm, Bregma 1.1–0.14 mm) mounted on SuperFrost + slides (Thermo Fisher Scientific, Reinach, Switzerland) were automatically immunostained using the Discovery XT technology (Ventana, Roche Diagnostics, Rotkreuz, Switzerland). Sections were de-paraffinized, rehydrated, subjected to antigen retrieval by heating with CC1 cell conditioning buffer for 28–68 min according to the antibody, then incubated for 1–3 h according to the primary antibody at room temperature with primary antibody diluted in antibody diluent (Ventana), incubated with the respective biotinylated secondary antibody diluted in antibody diluent, reacted with a DABMab kit (Ventana) and counterstained with Hematoxylin II and Bluing reagent (Ventana). Slides were washed with soap in hot tap water and rinsed under cold running tap water to remove the soap, then dehydrated and embedded with Pertex.

For luxol fast blue (LFB) staining, slides were de-paraffinized and rehydrated to 95% ethanol. Slides were then incubated in LFB solution [Solvent Blue 38 (Sigma S3382) in 95% ethanol and 10% acetic acid (Sigma 695092)] overnight at 60 °C, rinsed in 95% ethanol for 1 min, then in distilled water for 2 min and in 0.05% lithium carbonate (Merck 105680; Merck Millipore, Schaffhausen, Switzerland) for 5 s. Subsequently, slides were rinsed in 70% ethanol twice for 10 s, then in distilled water for 2 min. The rinsing was repeated in 0.05% lithium carbonate prepared freshly, 70% ethanol and distilled water until there was a sharp contrast between the blue of the white matter (myelin) and the colorless grey-matter. Finally, slides were dehydrated starting with 95% ethanol and mounted in Pertex.

### Antibodies

Primary antibodies were: rabbit anti-mouse myelin basic protein (MBP) (Dako A0623; Dako, Carpinteria, CA, USA) 1:1000; rabbit anti-mouse glutathione S-transferase-π (GST-π) (MBL 312; MBL International, Woburn, MA, USA) 1:500; Rabbit anti-Iba1 (Wako 019-19741, 50 μg/100 μl; Wako, Osaka, Japan) 1:500; rabbit anti-GFAP (glial fibrillary acidic protein, Dako Z0334) 1:5000; rabbit anti-dMBP (debris of MBP) (Merck Millipore AB5864) 1:3000; mouse anti-Neurofilament (Covance SMI312; BioLegend Covance, San Diego, CA, USA) 1:5000; rat anti-LAMP-1 (CD107a, Bio-Rad, Cressier, Switzerland, MCA4707T) 1:200. Rabbit anti-TMEM119 (209064, Abcam, Cambridge, UK).

Secondary detection antibodies were: Goat anti-rabbit IgG biotinylated (Jackson ImmunoResearch 111-065-144; Jackson ImmunoResearch, Cambridgeshire, UK) 1:1000; Goat anti-rabbit IgG biotinylated (Vector BA-1000; Vector Laboratories, Peterborough, UK) 1:200 or 1:1000; Goat anti-mouse IgG biotinylated (Vector BA-9200) 1:1000.

### Analysis of histological images

For the quantitative evaluation of microglia numbers and morphology, a proprietary image analysis platform (ASTORIA, Automated Stored Image Analysis, Novartis Pharma AG, Basel, Switzerland) was developed based on MS Visual Studio 2010 (Microsoft, Seattle, WA, USA) and many functions from Matrox MIL V9 libraries (Matrox Inc., Dorval, Quebec, Canada) as described elsewhere [28]. One to two coronal brain sections (from the preoptic area, approximate coordinates according to Paxinos and Franklin: interaural 4.9–3.94 mm, Bregma 1.1–0.14 mm) per animal were used for image analysis at the level of the corpus callosum (cc) and the external capsule (ec).

The following procedure was followed for the detection and analysis of soma, proximal and distal processes as described earlier [28]: (1) brain sections containing immunohistochemically stained microglia (Iba1) soma and their proximal and distal processes (all in brown) were scanned with Aperio’s Scanscope (Leica Biosystems, Wetzlar, Germany) at 20× magnification; and (2) Each image was processed using the ImageScope software (V12.1.0.5029, Aperio) according to the following steps: (A) color deconvolution to obtain brown staining without blue; (B) segmentation of brain tissue from white background through thresholding, morphological closing, filling of holes, opening and elimination of too small objects, resulting in a binary mask of the valid tissue and sample area; (C) adaptive thresholding for the individual segmentation of soma, based on the average gray value of the blue channel of the color-deconvoluted brown image at sufficiently dark regions (indicative for soma). The computed threshold was used for binarization, and after size filtering yielded the soma mask image (within the valid sample region); (D) segmentation of processes through morphological top hat transformation with a size to pick thin processes. Adaptive thresholding was applied again to segment the processes (using the previously determined gray average of brown objects), followed by binarization of the top hat image and size filtering of the resulting objects; (E) subtraction of soma (that may also have been picked by top hat thresholding) to obtain an image mask of true processes; (F) ultimate thinning of processes for length computation; (G) proximal processes: a predefined number of dilations of soma was used to define a reference (marker) region for proximal soma, employing a circle around the soma centre to define the cutoff boundary for proximal processes. Thinned proximal processes with marker in dilated soma and limited by circular influence zones (set of “proximal thinned processes”) were then reconstructed around the soma centre. “Final proximal processes” were collected through reconstruction of all processes having markers in the “proximal thinned processes” set; (H) soma was added to proximal processes to obtain a set of “visible microglia”; (I) distal processes: reconstruction of processes from proximal processes only (i.e., ignoring those in the background or from the soma in a different focus plane), then subtraction of circular regions defining proximal processes to yield a set of distal processes; (J) in the optical density computation for soma as well as “visible microglia” (individual soma + proximal processes complex within circular reference region), local background (non-visible microglia) was used as reference; (K) morphometric features (size, form factor, length) were computed for soma, proximal and distal processes. Microglia numbers (microglia soma numbers normalized to the region of interest) and microglia activation (Microglia and proximal processes area normalized to non-soma-associated processes area) were used as microglia readouts.

This image analysis algorithm was also used to quantify the stained areas of oligodendrocytes (GST-π) and astrocytes (GFAP) in coronal brain sections according to the above description. Luxol Fast Blue was analyzed by ASTORIA using the integrated optical density (IOD) parameter.

The HALO Image Analysis Platform (Indica Labs, Albuquerque, NM, USA) was used to analyze myelin debris (dMBP), lysosomal marker (LAMP-1), neurofilament (SMI312) and homeostatic microglia marker (TMEM119).

### Statistics

MRI data were analyzed using ANOVA with random effects (SYSTAT 13, SYSTAT Software, Inc., San Jose, CA, USA) to take the longitudinal character of the data into account. Histology and biochemical data were analyzed by Ordinary one-way ANOVA Holm–Sidak's multiple comparison tests or Holm–Sidak’s two-way ANOVA with multiple comparisons to compare effects within one genotype and to compare across genotypes at one timepoint as specified in the figure legends. Statistical analyses are specified in the figure legends if other methods were applied. Statistical significance was assumed for *p* < 0.05.

## Results

### TREM2-sol express soluble TREM2 only and lack cell surface TREM2 in opposition to TREM2-IPD showing increased cell surface expression

**Fig. 1:**
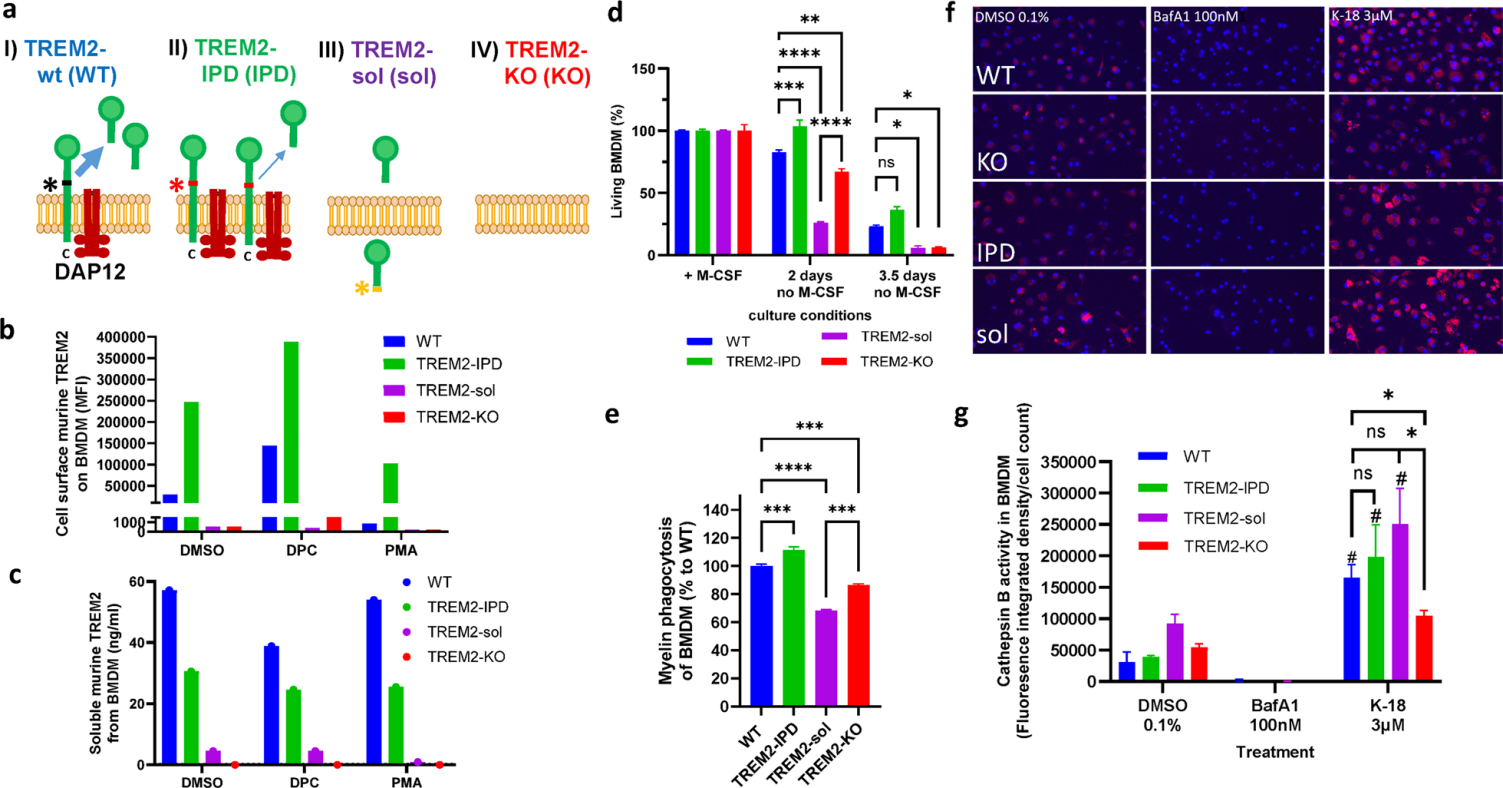
Characterization of BMDM from wild-type, TREM2-IPD, TREM2-sol and TREM2-KO mice. **a** Schematic representation of the TREM2 receptor. Black, red and yellow asterisks indicate the cleavage site in WT, the mutated cleavage site in TREM2-IPD and in TREM2-sol, respectively. **b** Flow cytometry analysis of murine cell surface TREM2 on BMDM. MFI: median fluorescence intensity. Sheddase inhibitor: DCP333 (DPC), sheddase activator PMA. **c** Analysis of supernatants from **b** of murine soluble TREM2 from BMDM. **d** ATP-based cell survival assay of BMDM upon M-CSF deprivation for 2 and 3.5 days. ATP levels of cells cultured with M-CSF (*n* = 7) were set as 100% survival and compared to the ATP concentration after 2 (*n* = 4) and 3.5 days (*n* = 3) without M-CSF for each genotype. Statistics: Holm–Sidak’s two-way ANOVA multiple comparisons (*p ≤ 0.05, **p ≤ 0.01, ***p ≤ 0.001, ****p ≤ 0.0001). **e** In vitro phagocytosis capacity of BMDM over 12 h (area-under-the curve) with 5 µg pHrodo-myelin per well (*n* = 3). Fluorescence measurements in wells without prey were used as controls (data not shown). **f** Representative images of the Cathepsin B activity assay taken by the In-Cell Analyzer. The nuclei are stained with DAPI (blue), and the red fluorescence signals are derived from cleaved Magic red. **g** Quantification of the Cathepsin B assay images. The fluorescence integrated density of the Magic red signal was measured and normalized to the nuclei count. A significant (*p* < 0.05) increase in normalized fluorescence between the DMSO control and K-18 within one genotype is marked by #. Statistics for **d** and **g**: Holm–Sidak’s two-way ANOVA with multiple comparisons (*p ≤ 0.05, **p < 0.01, ***p < 0.001, ***p < 0.0001). Statistics for **e**: One-way ANOVA test with Holm–Sidak’s multiple comparisons test (***p < 0.001; ****p < 0.0001). All data are presented as means ± SEM

Different TREM2 transgenic mice were generated to investigate the role of TREM2 in neuroinflammatory disease conditions, especially in myelination processes. For this, mice expressing cleavage-reduced TREM2 (TREM2-IPD, IPD) and soluble only TREM2 (TREM2-sol, sol) mice were established (Fig. 1a). TREM2-IPD mice, described in detail earlier [14], have been generated by introducing the IPD amino-acids into the cleavage site. TREM2-sol mice were generated by introducing a stop-codon (TGA) into the cleavage-site of TREM2 [3] via CRISPR-Cas9 (see “Materials and methods” section for details, Fig. 1a). Finally, mice lacking completely TREM2 (TREM2 knockout, TREM2-KO, KO) mice [29] as well as wild-type mice (TREM2-WT, WT) were included in the studies.

BMDM and primary microglia from TREM2-IPD, TREM2-sol, TREM2-KO and WT mice were isolated and characterized (Fig. 1 and Additional file 1: Fig. S1). BMDM of all four genotypes were differentiated into active macrophages with IL-4 and IL-13. FACS was used to investigate TREM2 cell surface expression on BMDM. TREM2 was not detected on the cell surface of TREM2-KO and TREM2-sol BMDM, while the amount of cell surface TREM2 on untreated TREM2-IPD BMDM was substantially higher than on untreated WT cells (Fig. 1b). As ADAM17 is the key sheddase for TREM2 [3], the effects of the ADAM17 inhibitor, DPC333 [36], and of Phorbol-12-myristate-13-acetate (PMA, sheddase inducing) were evaluated to probe TREM2. DPC333 (DPC) treatment increased TREM2 on the cell surface of both WT and TREM2-IPD BMDM. PMA caused a complete absence of TREM2 on WT cells, while cell surface TREM2 was still observed on TREM2-IPD cells compared to untreated BMDM (Fig. 1b) as shown earlier [14]. In TREM2-sol and TREM2-KO no cell-surface TREM2 could be detected in the treatment conditions. From the same experiment, supernatants were collected to measure soluble TREM2 (Fig. 1c). Soluble TREM2 levels in WT compared to TREM2-IPD are sheddase activity dependent, as a substantial reduction with the sheddase inhibitor DPC333 was visible in WT and much less in TREM2-IPD (Fig. 1c). PMA-induced sheddase activity did not obviously change soluble TREM2 levels compared to DMSO control (Fig. 1c). Taken together, soluble TREM2 levels were reduced in the cleavage-reduced TREM2-IPD as has been shown earlier as well [14]. Importantly, soluble TREM2 levels were highly reduced in BMDM from TREM2-sol mice compared to WT in all three conditions (Fig. 1c). In TREM2-sol BMDM no intracellular accumulation of soluble TREM2 was observed (Additional file 1: Fig. S1a). However, the TREM2 RNA levels in TREM2-sol brains were reduced compared to wild-type (Additional file 1: Fig. S1b) which could account partially for the observed reduced soluble TREM2 protein levels.

### TREM2-sol showed augmented reduction of cell survival compared to TREM2-KO, while it was increased in TREM2-IPD

The survival of BMDM upon M-CSF withdrawal was analyzed based on ATP quantification. After 2 days without M-CSF, significant differences in the survival rate were observed between all genotypes: while about 80% of WT, 70% of the TREM2-KO, and only 25% of TREM2-sol BMDM were still alive, TREM2-IPD cells were unaffected by M-CSF deprivation (Fig. 1d). The TREM2-IPD observation is consistent with an earlier report [14]. On day 3.5, the positive effect of TREM2 was obvious as well. With less than 10% of surviving cells, TREM2-KO and TREM-sol BMDM showed significantly lower survival than WT and TREM2-IPD cells after 3.5 days of M-CSF withdrawal (Fig. 1d). These data indicate that plasma-membrane TREM2 conveys a pro-survival signal in BMDM independent from the presence of M-CSF and that soluble TREM2, at the observed reduced levels, seems to further impair BMDM survival.

Interestingly, pro-inflammatory cytokine MIP-1β levels were elevated in the supernatant of TREM2-IPD BMDM, while no such increase was observed for the other genotypes (Additional file 1: Fig. S1c). This indicates sustained TREM2 activation in TREM2-IPD BMDM as described earlier [14].

### TREM2-sol showed reduced, TREM2-IPD enhanced phagocytosis, but, for both, no impairment of the endo-lysosomal pathway in stark contrast to TREM2-KO

The BMDM phagocytic activity in TREM2-KO and TREM2-sol was significantly reduced compared to that in cells from WT mice (Fig. 1e). In contrast, BMDM from TREM2-IPD showed an enhancement of myelin phagocytosis compared to WT (Fig. 1e) as described earlier for microglia [14]. Similar results for the four different genotypes were observed in myelin phagocytosis of primary microglia (Additional file 1: Fig. S1d).

Finally, the endo-lysosomal activity of WT, TREM2-IPD, TREM2-KO, and TREM2-sol BMDM was investigated using Magic red, a cell penetrant substrate that becomes fluorescent inside the cell in lysosomes upon enzymatic cleavage by Cathepsin B. Cells were either treated with BafA1, which inhibits the acidification of the lysosome, or K-18, which inhibits the fusion of the lysosome and the autophagosome. BafA1 treatment completely diminished all fluorescence signals (Fig. 1f, g). K-18 caused an intracellular accumulation of Magic Red (Fig. 1f, g) and a significant increase in the fluorescence integrated density per cell count compared to the DMSO control in all BMDM genotypes except for TREM2-KO (Fig. 1g). There were no significant differences in the endo-lysosomal activity between the genotypes in the DMSO control. However, under K-18 treatment TREM2-KO BMDM displayed a significantly lower endo-lysosomal activity than BMDM from the other genotypes (Fig. 1g). This suggests that the activity of the endo-lysosomal pathway was fully functional in WT, TREM2-IPD and TREM2-sol but not in TREM2-KO BMDM.

### MRI and histology revealed similar demyelination in all genotypes but TREM2-sol and TREM2-KO mice showed accumulation of myelin debris, lack of remyelination and enhanced axonal pathology with partial oligodendrocyte recovery in the acute cuprizone model

**Fig. 2:**
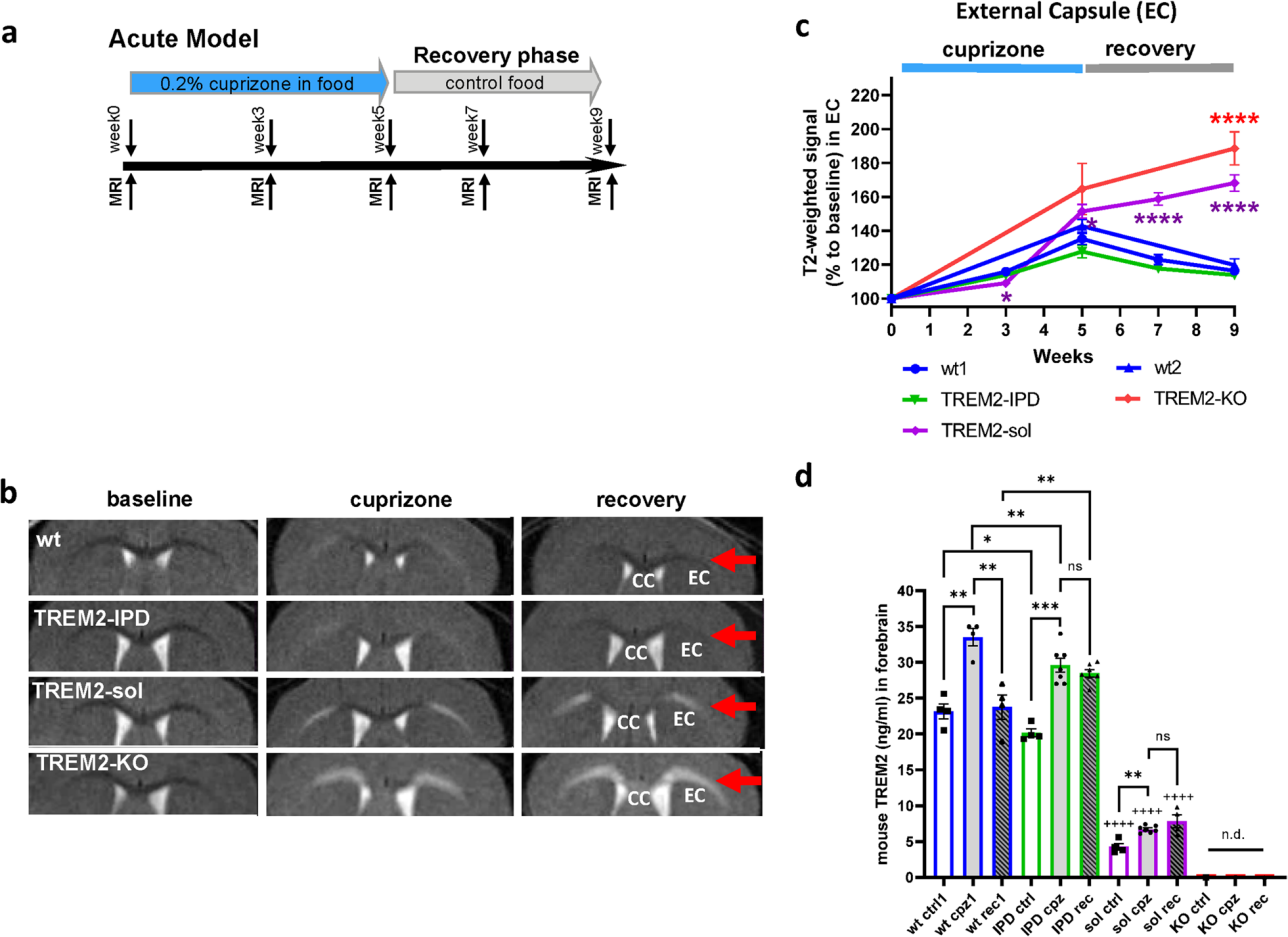
MRI indicated myelination deficits in TREM2-sol and TREM2-KO in the acute cuprizone model. **a** Schematic diagram of the experimental setup for the cuprizone treatment and recovery. Groups consisted of mice treated for 5 weeks with control food or 0.2% cuprizone in food and then switched back to control food (normal food) for the 4-week recovery. MRI measurements were performed at week 0 (baseline), week 3 (except for TREM2-KO and wt2) and week 5 of cuprizone intoxication, at week 7 (2 weeks of recovery on control food, except for TREM2-KO and wt2) and at week 9 (4 weeks of recovery on control food). Mice were culled at week 9 immediately after the last MRI measurement. **b** Representative MRI images acquired from three mice at baseline, at maximal pathology (5 weeks of receiving 0.2% cuprizone) and at recovery (4 weeks after switching to control food) for the different genotypes. **c** Corresponding T2-weighted MRI signal intensity (relative to the signal intensity at baseline) in external capsule (EC). Group sizes: *n* = 7–9 for all genotypes and timepoints. Data are shown as means ± SEM. Statistics: ANOVA with random effects comparisons indicated significant differences with respect to WT mice: *0.01 < *p* < 0.05, ***0.0001 < *p* < 0.001, ****p < 0.0001. For each group examined, T2-weighted signals were significantly increased with respect to baseline values (significances not shown). **d** Analysis of TREM2 levels in the brain for mice receiving control food (ctrl), at peak of cuprizone intoxication (week 5, cpz) and after 4-week recovery (rec). n.d. not detected. Statistics: Holm–Sidak’s multiple comparison test one-way ANOVA (*p < 0.05, **p < 0.01, ***p < 0.001, ^++++^*p* < 0.0001 to the respective wt group). Wt1, as well as wt ctrl1, wt cpz1 and wt rec1 are the respective wild-type groups for the study with TREM2-IPD and TREM2-sol, wt2 is the wild-type group for the TREM2-KO study

Cleavage-reduced TREM2 (TREM2-IPD), soluble TREM2 (TREM2-sol), TREM2-knockout (TREM2-KO) and wild-type TREM2 (wt) mice were treated for 5 weeks with 0.2% cuprizone delivered in food and then switched to normal food for 4 weeks (Fig. 2a). WT groups from the two independent studies (see Materials and methods) were plotted separately (wt1 for TREM2-IPD and TREM-sol; wt2 for TREM2-KO). Noninvasive, longitudinal magnetic resonance imaging (MRI) was performed at the beginning (baseline), at 3 and 5 weeks during cuprizone intoxication and at 7 and 9 weeks during recovery on normal food (Fig. 2a, b). Analysis of T_2_-weighted signal intensity is summarized for the EC (Fig. 2c) and the CC (Additional file 1: Fig. S2). During cuprizone treatment, MRI signal in the EC increased (Fig. 2b, c) indicating ongoing neuroinflammation and demyelination as demonstrated earlier for this model [28]. At week 3, no substantial genotype difference was obvious. At week 5 of cuprizone treatment, the T_2_-weighted signal intensity for TREM2-KO and TREM2-sol was slightly higher than for TREM2-IPD and WT animals (Fig. 2b, c). In WT and TREM2-IPD the MRI signal in EC decreased from weeks 5 to 9 during the recovery phase, while for TREM2-sol and TRE2-KO mice, it further increased despite discontinuation of cuprizone (Fig. 2c). TREM2-KO displayed an even stronger T_2_-weighted MRI signal intensity increase in CC which is quite different to TREM2-sol (Additional file 1: Fig. S2). In agreement with the T2-weighted signal results, MTR in the EC/CC of WT and TREM2-KO mice at week 5 was significantly lower than at baseline, and while a partial recovery of MTR occurred for WT animals from weeks 5 to 9 in the absence of cuprizone, a further reduction happened in TREM2-KO animals (Additional file 1: Fig. S3). The behavior of the T_2_-weighted signal was also reflected by an increase of the relaxation time T_2_ predominantly in the EC of cuprizone-challenged mice and by the sustained T_2_ increase in the EC throughout the recovery phase as exemplified for TREM2-sol mice (Additional file 1: Fig. S4). In addition, MTR reduction consistent with cuprizone-induced demyelination was detected in the CC and EC, but the reduction was more prominent in the EC (Additional file 1: Fig. S4). During the recovery phase, MTR in the EC of TREM2-sol mice remained low, while a partial recovery was seen in WT and TREM2-IPD animals (Additional file 1: Fig. S4).

Brain levels of soluble TREM2 for the four genotypes are summarized in Fig. 2d. At the peak of cuprizone intoxication soluble TREM2 was elevated in WT. TREM2 was also significantly elevated in TREM2-IPD animals, albeit to a lower extent indicating reduced TREM2 shedding. However, in the recovery phase at week 9 soluble TREM2 levels normalized in WT, while in TREM2-IPD, they remained elevated. In agreement with BMDM analyses (Fig. 1c), soluble TREM2 in TREM2-sol was reduced compared to WT mice but showed a significant increase (57%) upon cuprizone intoxication with no subsequent reduction during recovery. In TREM2-KO mice, TREM2 was consistently undetectable at all timepoints analyzed.

**Fig. 3:**
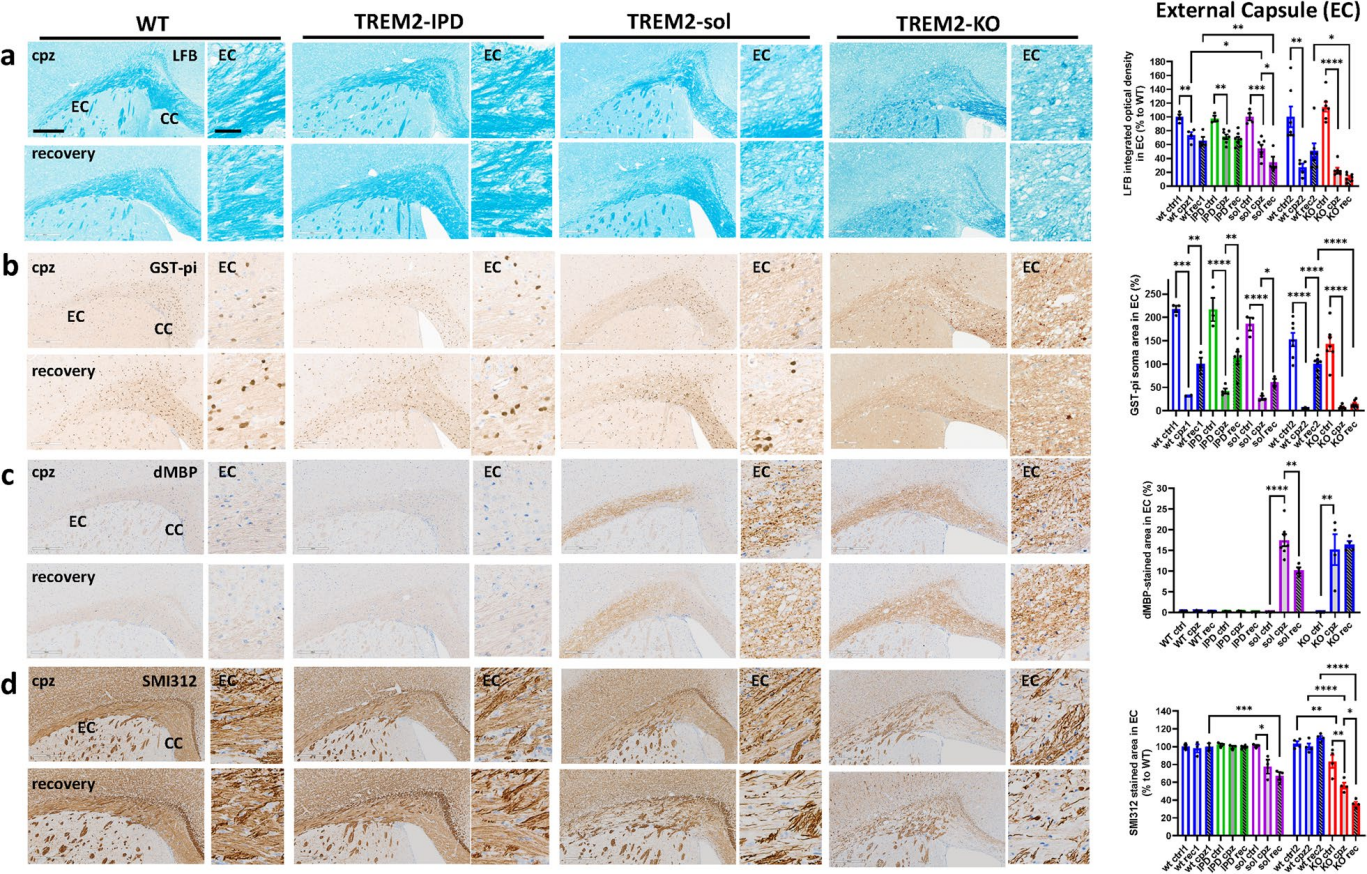
TREM2-sol and TREM2-KO display myelin debris, lack of remyelination and axonal pathology in the EC. Representative pictures for the different genotypes and timepoints from histological stainings detecting **a** myelin with Luxol Fast Blue (LFB) and corresponding quantitative optical density (OD) analysis of LFB in the EC (normalized to WT at control food), **b** mature oligodendrocytes (GST-π) and corresponding image analysis in EC (GST-π soma area in %), **c** myelin basic protein debris (dMBP) and corresponding image analysis in EC (dMBP-stained area in %), **d** neurofilament (SMI312) and corresponding image analysis in EC (SMI312-stained area in %). Group sizes: *n* = 3-7 for all genotypes and timepoints. Data shown as means ± SEM. wt: wild-type, TREM2-IPD: TREM2 cleavage-reduced, TREM2-sol: TREM2 soluble-only, TREM2-KO: TREM2 knockout. Ctrl: control food, cpz: cuprizone food for 5 weeks, rec: recovery on control food for 4 weeks. EC: external capsule, CC: corpus callosum. Scale bars: 300 µm (overview), 50 µm (close-up). Statistics: Holm–Sidak`s multiple comparison test one-way ANOVA (*p < 0.05, **p < 0.01, ***p < 0.001, ****p < 0.0001). Comparisons not indicated are non-significant. Wt ctrl1, wt cpz1 and wt rec1 are the respective wild-type groups for the study with TREM2-IPD and TREM2-sol, Wt ctrl2, wt cpz2 and wt rec2 are the wild-type groups for the TREM2-KO study. Only statistical analysis within a study was performed. For **c** the analysis of the respective wt group for TREM2-KO was omitted as no dMBP signal was observed

**Fig. 4:**
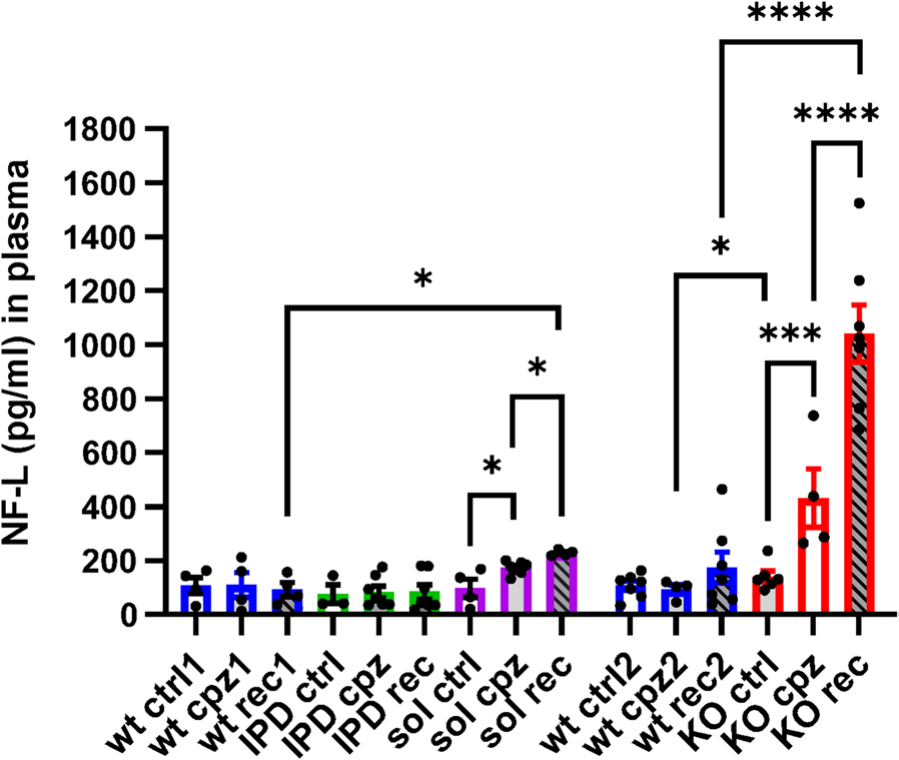
Increase of NF-L in plasma from TREM2-sol and TREM2-KO in the acute cuprizone model. NF-L measurements in plasma of WT, TREM2-IPD, TREM2-sol and TREM2-KO mice receiving control food (ctrl), at 5 weeks of cuprizone intoxication (cpz) and at 4-week recovery on normal food (rec). Group sizes: wt ctrl (*n* = 4), wt cpz (*n* = 4), wt rec (*n* = 4), TREM2-IPD ctrl (*n* = 3), TREM2-IPD cpz (*n* = 7), TREM2-IPD rec (*n* = 7), TREM2-sol ctrl (*n* = 4), TREM2-sol cpz (*n* = 7), TREM2-sol rec (*n* = 4), TREM2-KO cpz (*n* = 4). One-way ANOVA Holm–Šídák's multiple comparisons test, *p < 0.05, ***p < 0.001, ****p < 0.0001. Comparisons not indicated are non-significant. Wt ctrl1, wt cpz1 and wt rec1 are the respective wild-type groups for the study with TREM2-IPD and TREM2-sol, wt ctrl2, wt cpz2 and wt rec2 are the wild-type groups for the TREM2-KO study. Only statistical analysis within a study was performed

Cuprizone intoxication for 5 weeks resulted in a robust loss of myelin and oligodendrocytes in the EC and CC of all genotypes as revealed by LFB stain and GST-π immunohistochemistry (Fig. 3a, b and Additional file 1: Fig. S5). No obvious genotype-dependent difference in myelin and oligodendrocytes could be observed in animals receiving control food (Additional file 1: Fig. S6). In EC, the intensity of LFB stained myelin was reduced slightly stronger in TREM2-sol, while the extent was similar in TREM2-KO compared to the respective WT group (Fig. 3a). The number of GST-π-positive oligodendrocytes in EC, however, were reduced in all genotypes to the same degree (Fig. 3b). In the recovery phase at week 9 in EC no obvious remyelination in TREM2-IPD and WT mice could be observed, while myelination level were further reduced in TREM2-sol and TREM2-KO mice (Fig. 3a). On the contrary, GST-π-positive oligodendrocytes in the EC did substantially increase in TREM2-IPD and WT, while TREM2-sol mice showed a slight but significantly and TREM2-KO, as expected, no recovery of oligodendrocytes (Fig. 3b). This implicates that re-myelination in the EC at week 9 had not been taken place yet, despite some oligodendrocyte recovery. Importantly, TREM2-KO did not show any oligodendrocyte recovery in the EC at this timepoint. Staining against dMBP revealed no myelin debris in TREM2-IPD and WT mice at any timepoint (Fig. 3c). In addition, no myelin debris could be detected in any mouse receiving control food (Additional file 1: Fig. S6). Conversely, myelin debris was present in the EC of TREM2-sol and TREM2-KO animals during cuprizone intoxication and also in the recovery phase (Fig. 3c). The levels of dMBP were similar for both genotypes, TREM2-KO and TREM2-sol, at the peak of intoxication, while in the recovery phase dMBP levels reduced in TREM2-sol but not in TREM2-KO (Fig. 3c), implicating that some debris clearance capacity and resolution was present in TREM2-sol mice, which was also supported by the observed oligodendrocyte recovery. The presence of enhanced debris was paralleled by axonal pathology in the EC, characterized by a loss of neurite fibers stained with SMI312, especially in the recovery phase (Fig. 3d). The axonal pathology and fiber loss in the EC was higher and further aggravated in TREM2-KO compared to TREM2-sol (Fig. 3d). The observed axonal pathology was also obvious by the increased NF-L levels in plasma (Fig. 4). TREM2-sol mice showed only a slight but significant increase in plasma NF-L, whereas TREM2-KO displayed a more than threefold increase in the recovery phase. In contrast, no significant axonal pathology was obvious in TREM2-IPD and WT mice at any timepoint (Fig. 3d) as well as no change in plasma NF-L (Fig. 4).

Analogous findings were made in the CC (Additional file 1: Fig. S5), however, with some important differences. GST-π positive oligodendrocyte recovery in CC was as efficient in TREM2-KO and TREM2-sol as for TREM2-IPD and WT. Even a higher number of oligodendrocytes could be observed in the recovery phase in TREM2-sol which was paralleled by a slight but non-significant increase of myelination (LFB density). Again, myelin debris was observed in both TREM2-sol and TREM2-KO, but the levels in TREM2-KO were nearly 10× higher than that in TREM2-sol. Axonal pathology was also further aggravated in TREM2-KO compared to TREM2-sol.

Taken together, WT and TREM2-IPD displayed the same responses concerning de- and re-myelination processes in the acute cuprizone model. While demyelination was comparable, TREM2-sol and TREM2-KO showed an enhanced presence of myelin debris and axonal pathology in the EC and CC. Myelin debris and axonal pathology was sustained or even further dramatically aggravated in TREM2-KO compared to TREM2-sol. This in turn affected proper remyelination and oligodendrocyte restoration during the recovery phase, which was highly diminished in TREM2-KO. TREM2-sol mice showed brain region-specific differences in myelin and oligodendrocyte recovery, with no or minimal recovery in the EC, and robust recovery in the CC.

### TREM2-IPD as well as TREM2-sol displayed sustained neuroinflammation in the acute cuprizone model which was paralleled in TREM2-sol with an increase of both LAMP-1 and TMEM119 in contrast to TREM2-KO

**Fig. 5:**
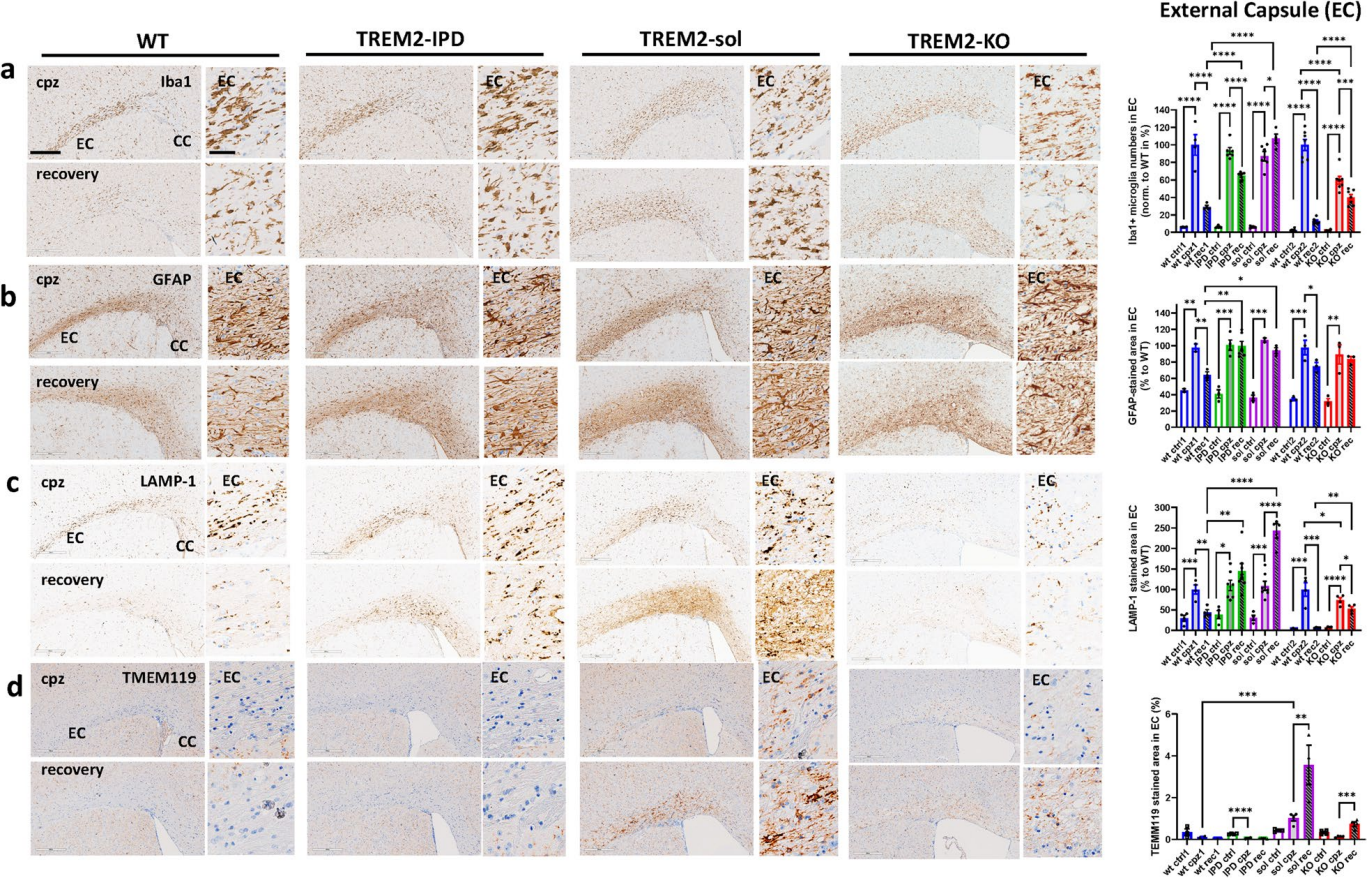
TREM2-IPD and TREM2-sol mice show both sustained microglia/astrocyte activation and enhanced LAMP-1 in the EC. Representative images for the different genotypes and timepoints from histological stainings detecting **a** Iba1 and corresponding image analysis of Iba1-positive soma numbers (normalized to WT at week 5 cuprizone), **b** astrocytes (GFAP) and corresponding image analysis (GFAP-stained area in %), **c** LAMP-1 (lysosomal-associated membrane protein 1) and corresponding image analysis (LAMP1-stained area in %), as well as **d** TMEM119 (homeostatic marker) and corresponding image analysis (TMEM119-stained area in %). Group sizes: *n* = 2-7 for all genotypes and timepoints. Data are shown as means ± SEM. WT: wild-type, TREM2-IPD: TREM2 cleavage-reduced, TREM2-sol: TREM2 soluble-only, TREM2-KO: TREM2 knockout. Ctrl: control food, cpz: cuprizone food for 5 weeks, rec: recovery on control food for 4 weeks. EC: external capsule. CC: corpus callosum. Scale bars: 300 µm (overview), 50 µm (close-up). Statistics: Holm–Sidak’s multiple comparison test one-way ANOVA (*p < 0.05, **p < 0.01, ***p < 0.001, ****p < 0.0001). Comparisons not indicated are non-significant. wt ctrl1, wt cpz1 and wt rec1 are the respective wild-type groups for the study with TREM2-IPD and TREM2-sol, wt ctrl2, wt cpz2 and wt rec2 are the wild-type groups for the TREM2-KO study. Only statistical analysis within a study was performed. For **d** the analysis of the respective wt group for TREM2-KO was omitted as no relevant TMEM119 signal was observed

**Fig. 6:**
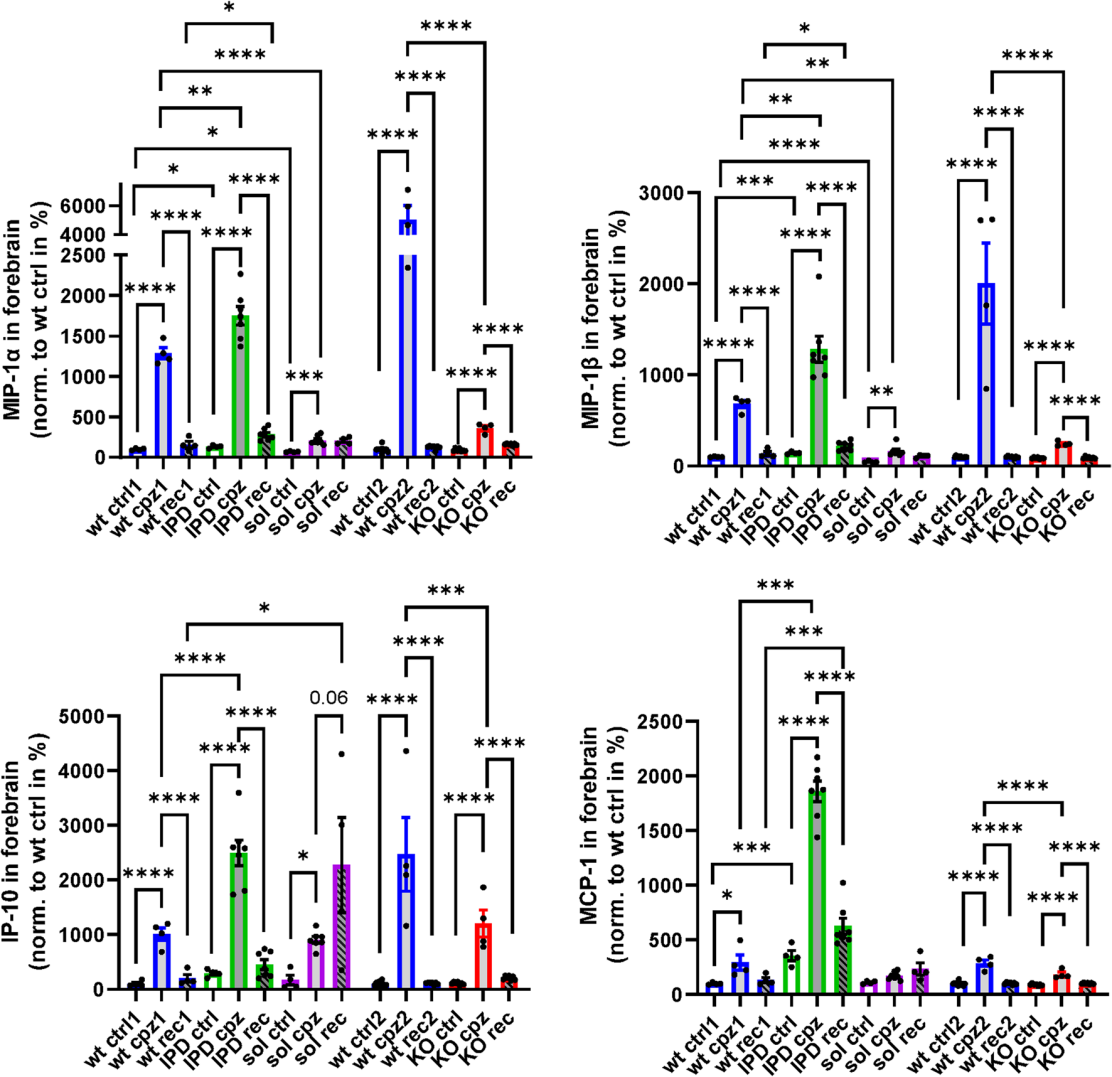
Brain cytokine/chemokine response is reduced in TREM2-sol and TREM2-KO, but enhanced in TREM2-IPD. Cytokine/chemokine measurements in brain detecting MIP-1a, MIP-1b, IP-10 and MCP-1 in WT, TREM2-IPD, TREM2-sol and TREM2-KO with control food (ctrl), at 5 weeks of cuprizone intoxication (cpz) and at 4-week recovery on normal food (rec). Measurements are normalized to wt cpz. Group sizes: wt ctrl (*n* = 4), wt cpz (*n* = 4), wt rec (*n* = 4), TREM2-IPD ctrl (*n* = 4), TREM2-IPD cpz (*n* = 7), TREM2-IPD rec (*n* = 7), TREM2-sol ctrl (*n* = 4), TREM2-sol cpz (*n* = 7), TREM2-sol rec (*n* = 4), TREM2-KO ctrl (*n* = 7), TREM2-KO cpz (*n* = 4), TREM2-KO rec (*n* = 7). Statistics: ordinary one-way ANOVA Holm–Šídák's multiple comparisons test, *p < 0.05, **p < 0.01, ***p < 0.001, ****p < 0.0001. Comparisons not indicated are non-significant. wt ctrl1, wt cpz1 and wt rec1 are the respective wild-type groups for the study with TREM2-IPD and TREM2-sol, wt ctrl2, wt cpz2 and wt rec2 are the wild-type groups for the TREM2-KO study. Only statistical analysis within a study was performed

Microglia in the EC and CC increased in numbers and became highly activated after cuprizone intoxication for 5 weeks (Fig. 5a, Additional file 1: Fig. S7). No increase in microglia was detected in any mouse receiving normal food throughout the study (Additional file 1: Fig. S6). In WT mice an expected increase of microglia numbers and activation could be observed in the EC upon 5-week cuprizone and a subsequent reduction of both parameters, albeit not to baseline levels, during the 4-week recovery phase on normal food (Fig. 5a, Additional file 1: Fig. S7). At the peak of cuprizone intoxication, TREM2-IPD as well as TREM2-sol showed a similar increase in microglia numbers and activation in the EC, while TREM2-KO displayed a smaller increase compared to the other genotypes (Fig. 5a, Additional file 1: Fig. S7). Interestingly, during the recovery phase microglia number and activation in the EC of TREM2-sol mice further increased, in contrast to the other genotypes (Fig. 5a). At week 5 of cuprizone challenge, GFAP-positive astrocytes in the EC were similarly increased for all genotypes (Fig. 5b). In the recovery phase, GFAP-positive astrocytes decreased for WT animals only, albeit not to baseline levels. Of note, the sustained neuroinflammation revealed by histology in TREM2-sol was not reflected in the cytokines/chemokines profile measured in the forebrain (Fig. 6). TREM2-sol, similar to TREM2-KO, showed only a slight increase of MIP-1α and MIP-1β that was significantly less than in WT at the peak of cuprizone intoxication (Fig. 6); the cuprizone-mediated increase for IP-10 and MCP-1 was less in TREM2-KO but comparable to WT in TREM2-sol. In the recovery phase IP-10 even further increased in only TREM2-sol, whereas in all other genotypes, IP-10 was back to baseline levels (Fig. 6). Finally, solely TREM2-IPD showed a dramatic increase of all measured cytokines/chemokines during cuprizone intoxication, while they decreased, but not to baseline levels (except for IP-10) during the recovery phase (Fig. 6).

Increases of the lysosome-associated membrane protein 1 (LAMP-1, CD107a) and of CD68 have been reported to be associated with microglia activation [8, 37]. Here, LAMP-1 expression during cuprizone intoxication increased to approximately the same levels in the EC of WT, TREM2-IPD and TREM2-sol mice; however, the increase was significantly less pronounced in TREM2-KO mice (Fig. 5c). Moreover, in the recovery phase the LAMP-1 staining intensity in the EC reduced in WT but not in TREM2-IPD mice, again pointing to a persistent microglia activation in the latter. In TREM2-sol LAMP-1 in the EC even further increased during recovery while displaying a rather diffuse and non-punctual staining (Fig. 5c). TMEM119, a homeostatic microglia marker which decreases during microglia activation [8], was very low in all genotypes in control condition (Additional file 1: Fig. S6) and no significant change could be observed in WT as well as TREM2-IPD mice in the acute cuprizone model at any timepoint (Fig. 5d, Additional file 1: Fig. S5). However, in TREM2-sol TMEM119 expression increased significantly in the EC upon cuprizone intoxication and it even further increased during the recovery period (Fig. 5d). In TREM2-KO only a slight increase of TMEM119 in the EC during recovery but not during cuprizone intoxication could be observed (Fig. 5d).

Similar findings to the EC were also made in the CC (Additional file 1: Figs. S5, S7). TREM2-IPD and TREM2-sol showed sustained microglia and astrocyte activation compared to WT. However, TREM2-KO displayed a further increase in microglia as well as astrocytes in the recovery phase in stark contrast to WT (Additional file 1: Figs. S5, S7). LAMP-1 was increased during recovery for both TREM2-IPD and TREM2-sol genotypes. During recovery TREM2-KO showed similar extent of increase in TMEM119 in the CC and EC; for TREM2-sol mice, TMEM119 was elevated in both regions; however, the increase was 4× higher in the EC than in the CC (Fig. 5c, Additional file 1: Fig. S5).

In summary, TREM2-IPD as well as TREM2-sol showed considerable sustained neuroinflammation and enhanced LAMP-1 during recovery in the acute cuprizone model. This was paralleled by an increase in cytokine/chemokines in the brain in TREM2-IPD, but much less in TREM2-sol. A further increase of TMEM119 as well as for LAMP-1 was observed in TREM2-sol. No substantial change of TMEM119 was observed in TREM2-IPD. TREM2-KO also displayed brain-region specific sustained microgliosis and no recovery of astrocytes, with a certain increase, albeit much less, for some cytokines/chemokines in the brain.

### Sustained TREM2 activation in TREM2-IPD led to enhanced myelination in the chronic cuprizone model

**Fig. 7:**
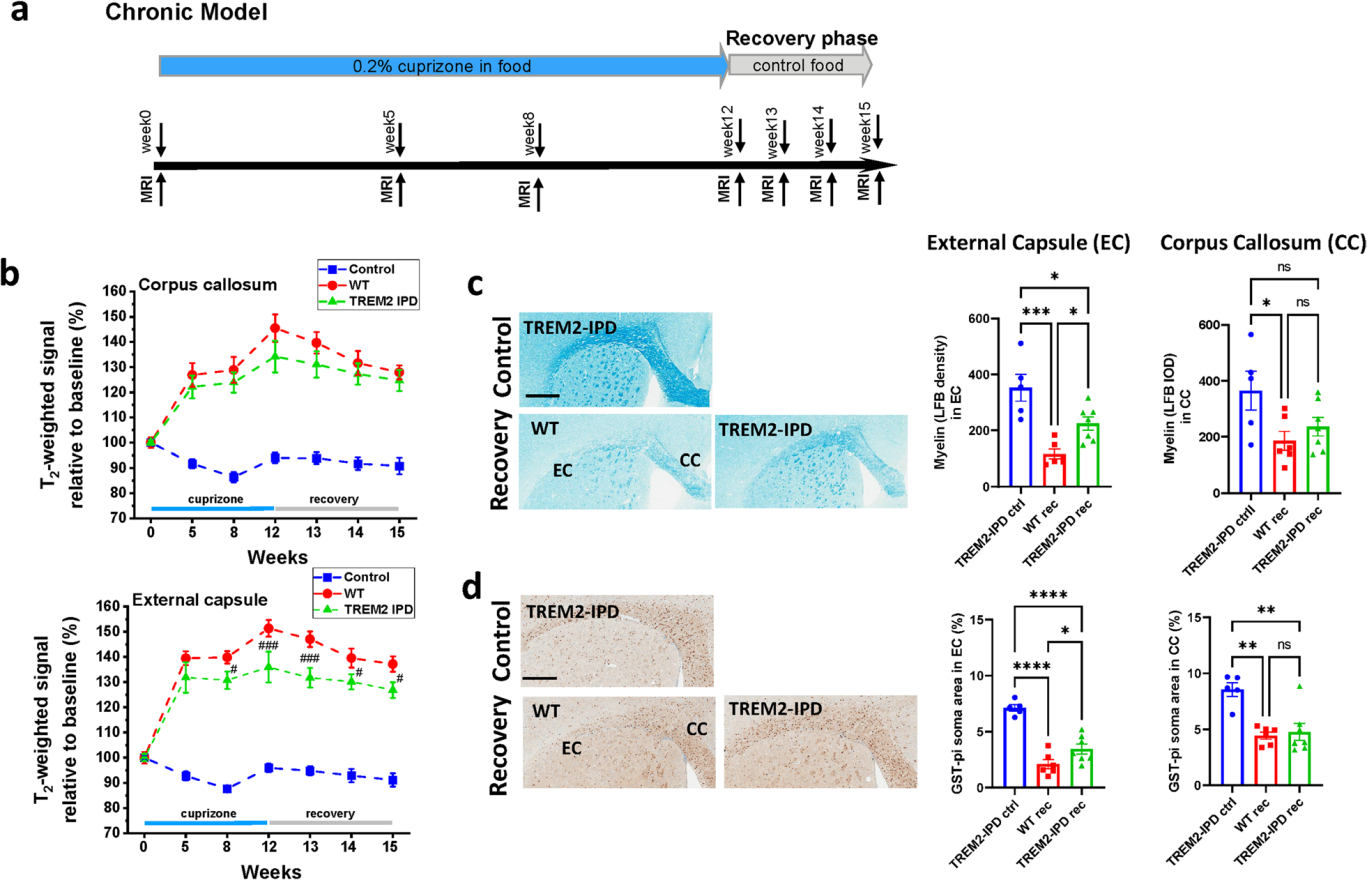
TREM2-IPD showed enhanced myelination in the chronic cuprizone model. **a** Schematic diagram of the experimental setup for the cuprizone treatment and recovery. Groups consisted of mice treated for 12 weeks with control food (normal food) or 0.2% cuprizone in food and then switched back to control food for the 3-week recovery. MRI measurements were performed at timepoints indicated. Mice were culled at week 15 immediately after the last MRI measurement. **b** T2-weighted signals in the CC and EC during the 12-week intoxication period and the recovery phase were significantly increased with respect to baseline values and compared to analyses for mice receiving control diet throughout the experiment. The significance levels ^#^0.01 < *p* < 0.05 and ^###^*p* < 0.001 correspond to ANOVA with random effects comparisons between WT and TREM2-IPD animals. Representative images for the different genotypes and at week 15 from histological stainings detecting **c** myelin with Luxol Fast Blue (LFB) and corresponding quantitative optical density analysis of LFB in the EC and CC, and **d** mature oligodendrocytes (GST-π) and corresponding image analysis in EC and CC (GST-π soma area in %). Group sizes: *n* = 5–7 for all genotypes and timepoints. Male mice were used for the cuprizone groups. Data are shown as means ± SEM. WT: wild-type, TREM2-IPD: TREM2 cleavage-reduced. Ctrl: control food, rec: recovery on control food for 3 weeks. Control refers to TREM2-IPD mice receiving normal food throughout the study. EC: external capsule, CC: corpus callosum. Scale bars: 500 µm. Statistics: ordinary one-way ANOVA Holm–Šídák’s multiple comparisons test, *p < 0.05, **p < 0.01, ***p < 0.001, ****p < 0.0001, ns: not significant

TREM2-IPD and WT mice were submitted to a chronic 12-week cuprizone intoxication followed by a 3-week recovery period (Fig. 7a) to analyze the involvement of sustained TREM2 activation under these harsher conditions. TREM2-sol and TREM2-KO were not included as substantial differences were already observed in the acute model (Fig. 2). Mice were taken down at the end of the recovery phase for histological analysis, and TREM2-IPD mice on normal food served as controls. For both genotypes, MRI revealed significantly enhanced T_2_-weighted signal in the EC and CC during the course of cuprizone intoxication and the recovery period (Fig. 7b), consistent with demyelination/neuroinflammation [28]. However, the signal increase in the EC was significantly lower for TREM2-IPD mice, whereas in the CC, there was a non-significant trend for this effect (Fig. 7b). Moreover, significantly increased relaxation time T_2_ values and significantly reduced MTR were detected in both the EC and CC in this chronic model, although no significant differences in these parameters were found for WT and TREM2-IPD mice (Additional file 1: Fig. S8). The in vivo observations were consistent with LFB staining at week 15 showing an increase of myelin in the EC in TREM2-IPD compared to WT (Fig. 7c). Similarly, an increased number of GST-π-positive oligodendrocytes in the EC could be observed after 3-week recovery in TREM2-IPD compared to WT (Fig. 7d). Microglia, astrocytes and LAMP-1 staining were similarly increased in the chronic cuprizone model in the recovery phase (Additional file 1: Fig. S9) as already observed in the acute model (Fig. 5). This indicates that under harsher and more chronic pathological conditions sustained TREM2 activation in TREM2-IPD mice led to beneficial effects on myelination processes.

## Discussion

TREM2 is essential for microglia to respond appropriately in certain disease conditions and fulfills homeostatic functions during development and aging. Human genetics revealed several mutations in TREM2 leading to a loss-of-function and to a disease called Nasu-Hakola which displays among others excessive central myelination defects [6]. Similarly, mice completely lacking TREM2 showed a dramatic impairment in microglia responses and myelination processes in the cuprizone model as previously described [21, 22, 38, 39]. Here, we confirm these findings in TREM2-KO mice in the 5-week cuprizone model by applying non-invasive longitudinal MRI measurements and different quantitative histological assessments. Furthermore, we report for the first time that mice expressing soluble-only TREM2 show similar pathology as TREM2-KO, but, nevertheless, also display explicit differences in microglia activation. We show that mice expressing soluble TREM2 display proper lysosomal function unlike macrophages from animals lacking completely TREM2. These novel mice, with a selective elimination of plasma membrane TREM2 and expressing exclusively soluble TREM2, albeit dramatically less than wild-type, were generated by introducing a stop codon into the cleavage site. So far, in humans, no variant is known that exclusively leads to soluble TREM2 expression, but a shedding enhancing cleavage site mutation has been described (H157Y) that is associated with AD increasing soluble TREM2 and reducing functional membrane TREM2 by approx. 50% [26]. Furthermore, alternative splicing events for TREM2 in the human brain have been described leading to either a soluble form or a TREM2 lacking the V-set immunoglobulin domain [40, 41]. In vitro characterization of BMDM from TREM2-sol mice confirmed lack of TREM2 cell surface expression, but with soluble TREM2 levels in the supernatant, although these were substantially lower than those found in WT animals. This might be in part due to the reduced TREM2 RNA levels observed in TREM2-sol brains (12 months of age), despite correct CRISPR/Cas9 modification as analyzed by TLA. Please note, the altered TREM2 RNA levels in TREM2-sol mice could also be due to differences in microglia proliferation and survival in the brain at this age which needs further evaluation. Furthermore, soluble TREM2 might get trapped and degraded in the lumen of the endoplasmic reticulum or Golgi or due to lack of hetero-dimerization with DAP12 is then not efficiently transported to the cell surface. Importantly, no intracellular accumulation was observed for soluble TREM2 in BMDM from TREM2-sol animals. In addition, in the present study, we confirmed earlier work [14] showing substantial phagocytosis increase in TREM2-IPD primary microglia using myelin as a prey. An increase in the phagocytic capacity, again using myelin as a prey, were also detected in BMDM from TREM2-IPD. More detailed analyses of the phagocytotic capacity of both cell types for other preys would be warranted but were out of the scope of the current work. Finally, sustained TREM2 activation in TREM2-IPD [14] behave very similar to WT mice in the acute but show enhanced remyelination and persistent microglia activation in the chronic cuprizone model.

MRI provided the opportunity to analyze non-invasively demyelination/remyelination processes in several brain areas in the cuprizone model. We had demonstrated earlier the significant correlation/negative correlation between T_2_-weighted signal intensity/MTR and histological parameters of myelin content in the cuprizone model [28]. Reduced myelin content is reflected in a reduced MTR, respectively, in an increased signal intensity. Consistent with the validation work performed earlier [28], upon acute cuprizone administration there was a significant T_2_-weighted signal increase and MTR reduction in the EC and CC for all genotypes. When cuprizone was discontinued at week 5, there was a partial reduction of T_2_-weighted signal/increase of MTR for WT and TREM2-IPD mice, in agreement with partial remyelination at week 9 evidenced histologically. For TREM2-sol and TREM2-KO animals the signal intensity further increased and MTR further reduced despite cuprizone discontinuation. Based on previous studies in the same model [28] and histological analysis carried out here, the higher MRI signal intensity for TREM2-sol and TREM2-KO mice might be related to debris accumulation and progression of neuroinflammation despite the interruption of the cuprizone treatment. Of note, at week 5 of cuprizone administration, homozygous TREM2-KO mice displayed a lower level of MTR reduction and a higher signal intensity compared to the other genotypes. Analogous results were obtained for the EC of TREM2-sol mice. Histology revealed the appearance of myelin debris during intoxication as well as its persistence in the recovery phase in the EC and CC of TREM2-KO and particularly in the EC of TREM2-sol mice. The discrepancy between MRI signal and MTR at week 5 for these animals might be linked to poor myelin debris removal, indicating that myelin debris also contributes to MTR and stressing the importance of assessing both parameters. This had already been pointed out earlier for the prophylactic treatment of WT mice with the colony stimulating factor 1 receptor (CSF1R) kinase inhibitor, BLZ945, in the same cuprizone model [28]: differences between MRI signal intensity and MTR changes could be explained by the accumulation of debris in the EC. These observations are consistent as well with the fact that oligodendrocyte ablation was reported to result only in minor MTR decrease due to inefficient removal of myelin debris [42]. During chronic cuprizone dosing for 12 weeks, T_2_-weighted signal in the EC and CC was increased with respect to baseline values and significantly lower in the EC of TREM2-IPD compared to WT mice. Histology at week 15 demonstrated a higher myelin content in the EC of TREM2-IPD mice. MTR was reduced in both the EC and CC with respect to baseline throughout the experiment, but it did not display significant differences between TREM2-IPD and WT animals despite being larger for TREM2-IPD at all timepoints measured. This suggests a lower sensitivity of MTR compared to T_2_-weighted signal analyses to follow pathology in this model.

There is strong evidence that microglia are vital for normal myelinogenesis, for clearance of myelin debris and modulation of oligodendrocyte precursor cell differentiation [18, 23, 43–46]. The inefficient clearance of myelin debris is, amongst other factors, responsible for an inefficient remyelination [21, 22, 38, 39]. During the recovery phase following chronic cuprizone dosing sustained microglia activation via cleavage-reduced TREM2 resulted in enhanced remyelination in the EC in TREM2-IPD mice. Analogously, enhanced microglia activation in TREM2-IPD, as observed by increased microglia numbers and activation as well as brain cytokines/chemokines, led to similar remyelination levels as in WT during the recovery phase after 5 weeks of cuprizone intoxication. This could be explained by the endogenous repair mechanisms that are able to respond in this acute model quite readily [17, 18]. Furthermore, the elevated astrocyte levels and activation in TREM2-IPD mice during the recovery could also have contributed to an augmented remyelination process [19, 47].

In the acute cuprizone model, similar pathology was observed in the EC of TREM2-sol mice expressing only the soluble form of TREM2 and in mice completely lacking TREM2; remyelination was impaired and axonal pathology was apparent. TREM2-sol as well as TREM2-KO animals displayed massive appearance of myelin debris in the EC, which hampered proper remyelination processes [48, 49]. Interestingly, in TREM2-sol mice microglia were induced by cuprizone, translated into increased numbers and activation, but were not capable of removing myelin debris. Indeed, in the present study microglia in TREM2-sol and TREM2-KO seemed to be dysfunctional and could not properly respond to the insult as indicated by the lack of some cytokine/chemokine induction as well as by the increased expression of the homeostatic marker, TMEM119 [8, 21, 50]. Surprisingly, BMDM survival was dramatically reduced for TREM2-sol compared to TREM2-KO animals. This is contrasting the microglia findings in the cuprizone model. Whereas TREM2-KO showed reduced microglia upon intoxication as expected, TREM2-sol mice displayed a rather enhanced microglia presence. This discrepancy is not understood and puzzling but could point to major differences between macrophages and microglia [51] and would need further characterization of both cell types but which is beyond the scope of this work. Astrocytes, which were highly induced in the EC of TREM2-sol and TREM2-KO mice, have been hypothesized to cause detrimental effects on myelination, depending on the pathology context [47]. It also has been shown that astrocytes crosstalk to microglia and support proper microglia function [18, 19, 52]. Finally, during cuprizone intoxication levels of LAMP-1, a phagolysosome marker that is upregulated in highly phagocytic microglia [50, 53] as well as in astrocytes [47], were elevated in the EC of TREM2-sol and comparable to those observed in the WT and TREM2-IPD animals. LAMP-1 induction was further increased in the recovery phase in TREM2-sol in stark contrast to TREM2-KO. Thus, it seems that TREM2-sol mice could upregulate the lysosomal pathway. This is further evidenced in BMDM from TREM2-sol that showed a robust cathepsin B activity that was similar to WT. However, BMDM lacking completely TREM2 were less efficient in regulating lysosomal function as shown here by reduced cathepsin B activity and also shown earlier [54]. Moreover, BMDM lacking TREM2 displayed inefficient intracellular degradation pathways, whereas soluble TREM2 was involved in regulating proper lysosomal pathway. Importantly, phagocytosis in BMDM and microglia from TREM2-sol and TREM2-KO was reduced, implicating that functional cell surface TREM2 is essential in this process as reported by other groups [22, 39]. However, it has been also shown that intracellular myelin clearance capability and not phagocytosis is reduced in cells lacking TREM2 [21], which we could partially confirm in vitro and in vivo. The rather diffuse LAMP-1 staining in TREM2-sol mice during recovery could also indicate a loss of proper LAMP-1 localization in microglia/astrocytes and thus gradually leading to lysosomal dysfunction in a more challenged context. This needs further investigation. Improper LAMP-1 localization has also been observed in other neurological diseases [55, 56]. Although less pronounced, some axonal loss was observed in TREM2-sol compared to TREM2-KO mice, and the increase in LAMP-1 could indicate more neuronal stress, which was also suggested by an increase in NF-L in plasma. Nevertheless, the persistent microglia activation, the increased LAMP-1 induction paralleled by a massive increase of TMEM119 and substantially reduced phagocytic activity in TREM2-sol indicate that soluble TREM2 is involved in regulating, albeit improperly, microglia. Similarly, it has been shown that soluble TREM2 enhances microglial inflammatory responses [57]. However, in contrast to our results it has been reported that soluble TREM2 protected against amyloid-beta-driven pathology in an Alzheimer’s disease model [58], and enhanced levels of soluble TREM2 in the cerebrospinal fluid were shown to correlate with an attenuated clinical progression in Alzheimer’s patients [59]. This difference to the cuprizone model could be due to the more chronic Alzheimer’s model and the expression of higher amounts of soluble TREM2 via an adeno-associated virus [58]. Overall, the reduced levels of soluble TREM2 detected in the forebrain of TREM2-sol mice might not have been sufficient to induce positive outcomes. Finally, the choice of the model used could also be important to decipher the directionality of TREM2-dependent effects [56]. For instance, the beneficial effect of cleavage-reduced TREM2 in TREM2-IPD mice was observed here in the chronic cuprizone model. In contrast, enhanced pathology of cleavage-reduced TREM2 was observed in an Alzheimer’s model [14]. Further studies are needed to unravel the TREM2-dependent cellular processes in different preclinical models.

Regional differences of remyelination and neuroinflammation were observed in the acute cuprizone model. While microglia numbers decreased during recovery in the EC of TREM2-IPD, they rather increased in the CC of the same animals. This was similar to TREM2-KO; however, in TREM2-sol mice, microglia increase was observed in both regions. Interestingly, a similar brain region-specific finding on microglia was observed when mice were prophylactically treated with the CSF1R kinase inhibitor, BLZ945, in the acute cuprizone model [28]. While microglia were depleted in the CC by BLZ945, microglia specifically in the EC were insensitive to treatment and still remained. An interaction of TREM2 and CSF1R has been recently hypothesized [60] indicating a convergence of both pathways leading to similar changes. Furthermore, it is now well-established that regional heterogeneity of microglia exists, e.g., on their transcriptional profile, functionality and number [50, 61–63]. Furthermore, our data suggest that there was a direct correlation between the amount of myelin debris and the improper microglia activation in the EC of TREM2-sol mice. This in turn lead to a lack of remyelination or even to further demyelination despite some recovery of oligodendrocytes. Of note, the amount of myelin debris in the CC of TREM2-sol mice was significantly smaller than that quantified in the EC. Given the fact that improper microglia activation was seen in both brain areas, it is reasonable to conclude that the oligodendrocytes recovery and ability to remyelinate in the CC of TREM2-sol animals was due to the smaller amount of debris in this region compared to the EC. In contrast, in TREM2-KO oligodendrocytes were not capable of promoting remyelination in the CC during the recovery phase, although improper microglia activation was similar to that in TREM2-sol. It has been shown that brain-region specific non-myelinating oligodendrocyte recovery can occur despite massive presence of myelin debris [64, 65] and this again calls for a careful analysis of regional differences and regional cell population heterogeneities.

In summary, our results further strengthen the role of TREM2 in proper microglia function towards brain insults, in particular myelin damage. We show for the first time that sustained TREM2 activation enhances remyelination in the chronic cuprizone model, whereas no such enhancement was observed in the acute paradigm. Furthermore, we show that soluble TREM2, even at lower levels, is involved in regulating lysosomal function, while functional cell-surface TREM2 is indispensable for myelin debris phagocytosis and finally remyelination. These results put TREM2 in the spotlight as an interesting target to modulate microglia and myelination processes in different demyelinating diseases.

## Supplementary Information

**Additional file 1: Figure S1.** Characterization of TREM2-sol in bone-marrow derived macrophages, brain and primary microglia. **a** Flow cytometry analysis of murine intracellular TREM2 in BMDM. MFI: median fluorescence intensity. Cells were either untreated or treated with the sheddase inhibitor DCP333 (5 µM overnight, DPC) or the sheddase activator PMA (50 ng/ml for 30 min). The gate for TREM2 positive cells was set based on the isotype control (threshold 0.2% positive cells in the isotype control). The dotted line marks the background signal which is observed in TREM2-KO. **b** Mouse Trem2 qRT-PCR of total RNA from WT and TREM2-sol brains (age 12 months). Normalized to Gapdh, 2^-ΔΔCt = 2^-(ΔCtTrem2-AvgΔCtControl/). n = 6 per group. Statistics: unpaired *t* test two-tailed. **: p < 0.01. **c** MIP1-β analysis in supernatants from BMDM for each genotype data in triplicates (n = 3) at days in vitro (DIV) 7. **d** In vitro phagocytosis of primary microglia over 24 h with 10 µg pHrodo-myelin per well. The WT AUC of the integrated fluorescence intensities was set to 100%. All genotypes were compared to the other three, and all significant differences are displayed. In both experiments, data for each genotype data were generated in triplicates (n = 3). Fluorescence measurements in wells without prey were used as controls (data not shown). Statistics: one-way ANOVA test with Tukey`s multiple comparisons test (***: p < 0.001, ****: p < 0.0001). **Figure S2.** T_2_-weighted MRI signal analysis in the CC region for the 5-week cuprizone model in WT, TREK2-KO, TREM2-sol and TREM2-IPD mice. Groups consisted of mice treated for 5 weeks with control food (normal food) or 0.2% cuprizone in food and then switched back to control food (normal food) for the 4-week recovery. MRI measurements were performed at week 0 (baseline), week 3 and week 5 of cuprizone intoxication, at week 7 (2 weeks of recovery on control food) and at week 9 (4 weeks of recovery on control food). Mice were culled at week 9 immediately after the last MRI measurement. EC: external capsule, CC: corpus callosum. Group sizes: n = 7–9 for all genotypes and at all timepoint. Statistics: ANOVA with random effects comparisons indicated significant differences with respect to WT mice: *: p < 0.05, ****: p < 0.0001. For each group examined, T_2_-weighted signals were significantly increased with respect to baseline values (significances not shown). Data are shown as means ± SEM. wt1 is the respective wild-type group for the study with TREM2-IPD and TREM2-sol, wt2 is the wild-type group for the TREM2-KO study. **Figure S3.** MTR analyses in the combined external capsule (EC) and corpus callosum (CC) region for the 5-week cuprizone and 4-week recovery model. Data (means ± SEM) presented as relative to baseline values for each genotype. Group sizes: n = 14 until week 5, n = 7 until week 9 for all genotypes. Statistics: ANOVA with random effects. The levels of significance ###: p < 0.001 correspond to comparisons betweenTREM2-KO and WT animals at the specified timepoints. WT: wild-type, TREM2-KO: TREM2 knockout. **Figure S4.** MRI analyses in external capsule (EC) and corpus callosum (CC) of TREM2-IPD and TREM2-sol mice for the 5-week cuprizone and 4-week recovery model. Data presented as means ± SEM. Group sizes: n = 7 for each genotype. Statistics: ANOVA with random effects. The levels of significance #: p < 0.05, ##: p < 0.01, ###: p < 0.001 correspond to comparisons betweenTREM2-sol and WT animals at the specified timepoints. WT: wild-type, TREM2-IPD: TREM2 cleavage-reduced, TREM2-sol: TREM2 soluble-only. **Figure S5.** Quantitative image analysis of histological stainings in CC. Luxol fast blue (LFB) optical density (OD) analysis of LFB (normalized to WT at control food), mature oligodendrocytes (GST-π soma area in %), myelin basic protein debris (dMBP-stained area in %), neurofilament (SMI312-stained area in %). Iba1-positive soma numbers (normalized to WT at week 5 cuprizone), LAMP-1 (lysosomal-associated membrane protein 1)-stained area in %, TMEM119 (homeostatic marker, TMEM119-stained area in %), astrocytes (GFAP-stained area in %). Group sizes: n = 2-7 for all genotypes and timepoints. Data shown as means ± SEM. WT: wild-type, TREM2-IPD: TREM2 cleavage-reduced, TREM2-sol: TREM2 soluble-only, TREM2-KO: TREM2 knockout. Ctrl: control food, cpz: cuprizone food for 5 weeks, rec: recovery on control food for 4 weeks. CC: corpus callosum. Scale bars: 300 µm (overview), 50 µm (close-up). Statistics: Holm–Sidak`s multiple comparison test one-way ANOVA (*: p < 0.05, **: p < 0.01, ***: p < 0.001, ****: p < 0.0001). Comparisons not indicated are non-significant. Wt ctrl1, wt cpz1 and wt rec1 are the respective wild-type groups for the study with TREM2-IPD and TREM2-sol, wt ctrl2, wt cpz2 and wt rec2 are the wild-type groups for the TREM2-KO study. Only statistical analysis within a study was performed. The analyses of dMBP and TMEM119 for the respective wild-type groups for TREM2-KO were omitted as no obvious signals were observed. **Figure S6.** Brain histological analysis for WT, TREM2-IPD, TREM2-sol and TREM2-KO mice receiving control normal food showed no difference between groups. Quantitative image analysis is shown in Figs. 3 and 5. Luxol Fast Blue (LFB), mature oligodendrocytes (GST-π), myelin basic protein debris (dMBP), neurofilament (SMI312), Iba1, LAMP-1 (lysosomal-associated membrane protein 1), TMEM119 (homeostatic marker), astrocytes (GFAP). WT: wild-type, TREM2-IPD: TREM2 cleavage-reduced, TREM2-sol: TREM2 soluble-only, TREM2-KO: TREM2 knockout. ec: external capsule, cc: corpus callosum. Scale bars: 300 µm. **Figure S7.** Image analysis of Iba1-positive microglia activation in the EC and CC (normalized to WT in %) from the staining shown in Fig. 5. Microglia activation is calculated as microglia and proximal processes area normalized to non-soma-associated processes area. Data are shown as means ± SEM. WT: wild-type, TREM2-IPD: TREM2 cleavage-reduced, TREM2-sol: TREM2 soluble-only, TREM2-KO: TREM2 knockout. Ctrl: control food, cpz: cuprizone food for 5 weeks, rec: recovery on control food for 4 weeks. ec: external capsule, cc: corpus callosum. Statistics: Holm–Sidak`s multiple comparison test one-way ANOVA (*: p < 0.05, **: p < 0.01, ***: p < 0.001, ****: p < 0.0001). Comparisons not indicated are non-significant. wt ctrl1, wt cpz1 and wt rec1 are the respective wild-type groups for the study with TREM2-IPD and TREM2-sol, wt ctrl2, wt cpz2 and wt rec2 are the wild-type groups for the TREM2-KO study. Only statistical analysis within a study was performed. **Figure S8.** MRI analyses of TREM2-IPD mice in the chronic cuprizone model. Data presented as means ± SEM. Group sizes: n = 7 for each genotype. Statistics: ANOVA with random effects. The levels of significance #0.01 < p < 0.05, ##0.001 < p < 0.01 correspond to comparisons between TREM2-IPD and WT animals at the specified timepoints. WT: wild-type, TREM2-IPD: TREM2 cleavage-reduced. Control refers to TREM2-IPD mice receiving normal food throughout the study. **Figure S9.** Microglia (Iba1), astrocytes (GFAP) and lysosomal marker LAMP-1 were quantified in WT and TREM2-IPD in the chronic cuprizone model (12-week cuprizone treatment and 3-week recovery). Group sizes: n = 5–7. Data are shown as means ± SEM. WT: wild-type, TREM2-IPD: TREM2 cleavage-reduced. Ctrl: control food, rec: recovery on control food for 3 weeks. EC: external capsule, CC: corpus callosum. Scale bars: 500 µm. Statistics: Holm–Sidak`s multiple comparison test one-way ANOVA (*: p < 0.05, **: p < 0.01, ****: p < 0.0001, n.s.: not significant).

## Data Availability

All data generated or analyzed during this study are included in this published article.

## Abbreviations

cc: Corpus callosum
ec: External capsule
GFAP: Glial fibrillary acidic protein
GST-π: Glutathione *S*-transferase-π
KO: Knockout
LFB: Luxol fast blue
MBP: Myelin basic protein
dMBP: Debris of MBP
MRI: Magnetic resonance imaging
ms: Millisecond
MTR: Magnetization transfer ratio
SEM: Standard error of the mean
TREM2: Triggering receptor expressed on myeloid cells 2
WT: Wild-type
