# Supplementary material for: Genetic models of cleavage-reduced and soluble TREM2 reveal distinct effects on myelination and microglia function in the cuprizone model
Source: J Neuroinflammation. 2023 Feb 8;20:29. doi: 10.1186/s12974-022-02671-z (PMC9909920; doi:10.1186/s12974-022-02671-z)
Supplement: Supplementary file 1 — Additional file 1: Figure S1. Characterization of TREM2-sol in bone-marrow derived macrophages, brain and primary microglia. a Flow cytometry analysis of murine intracellular TREM2 in BMDM. MFI: median fluorescence intensity. Cells were either untreated or treated with the sheddase inhibitor DCP333 (5 µM overnight, DPC) or the sheddase activator PMA (50 ng/ml for 30 min). The gate for TREM2 positive cells was set based on the isotype control (threshold 0.2% positive cells in the isotype control). The dotted line marks the background signal which is observed in TREM2-KO. b Mouse Trem2 qRT-PCR of total RNA from WT and TREM2-sol brains (age 12 months). Normalized to Gapdh, 2^-ΔΔCt = 2^-(ΔCtTrem2-AvgΔCtControl/). n = 6 per group. Statistics: unpaired t test two-tailed. **: p < 0.01. c MIP1-β analysis in supernatants from BMDM for each genotype data in triplicates (n = 3) at days in vitro (DIV) 7. d In vitro phagocytosis of primary microglia over 24 h with 10 µg pHrodo-myelin per well. The WT AUC of the integrated fluorescence intensities was set to 100%. All genotypes were compared to the other three, and all significant differences are displayed. In both experiments, data for each genotype data were generated in triplicates (n = 3). Fluorescence measurements in wells without prey were used as controls (data not shown). Statistics: one-way ANOVA test with Tukey`s multiple comparisons test (***: p < 0.001, ****: p < 0.0001). Figure S2. T2-weighted MRI signal analysis in the CC region for the 5-week cuprizone model in WT, TREK2-KO, TREM2-sol and TREM2-IPD mice. Groups consisted of mice treated for 5 weeks with control food (normal food) or 0.2% cuprizone in food and then switched back to control food (normal food) for the 4-week recovery. MRI measurements were performed at week 0 (baseline), week 3 and week 5 of cuprizone intoxication, at week 7 (2 weeks of recovery on control food) and at week 9 (4 weeks of recovery on control food). Mice were culled at week 9 immediatel [file 12974_2022_2671_MOESM1_ESM.pdf]

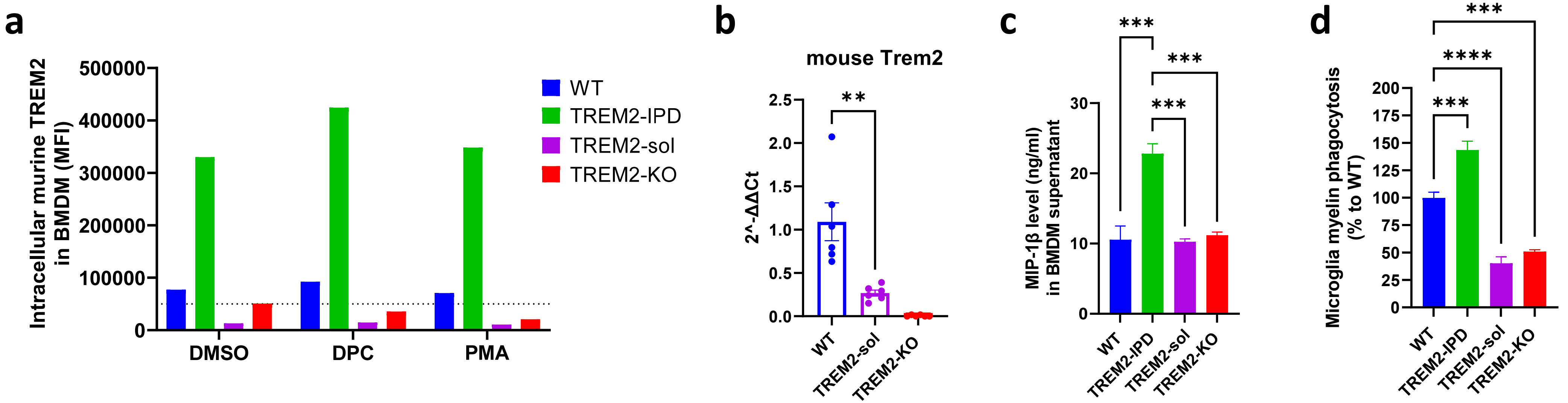

a
Intracellular murine TREM2 in BMDM (MFI)
500000
400000
300000
200000
100000
0
DMSO
DPC
PMA
WT
TREM2-IPD
TREM2-sol
TREM2-KO
b
mouse Trem2
2^-ΔΔCt
2.5
2.0
1.5
1.0
0.5
0.0
**
WT
TREM2-sol
TREM2-KO
c
MIP-1β level (ng/ml) in BMDM supernatant
30
20
10
0
***
***
***
WT
TREM2-IPD
TREM2-sol
TREM2-KO
d
Microglia myelin phagocytosis (% to WT)
200
175
150
125
100
75
50
25
0
***
****
***
WT
TREM2-IPD
TREM2-sol
TREM2-KO

Figure S1

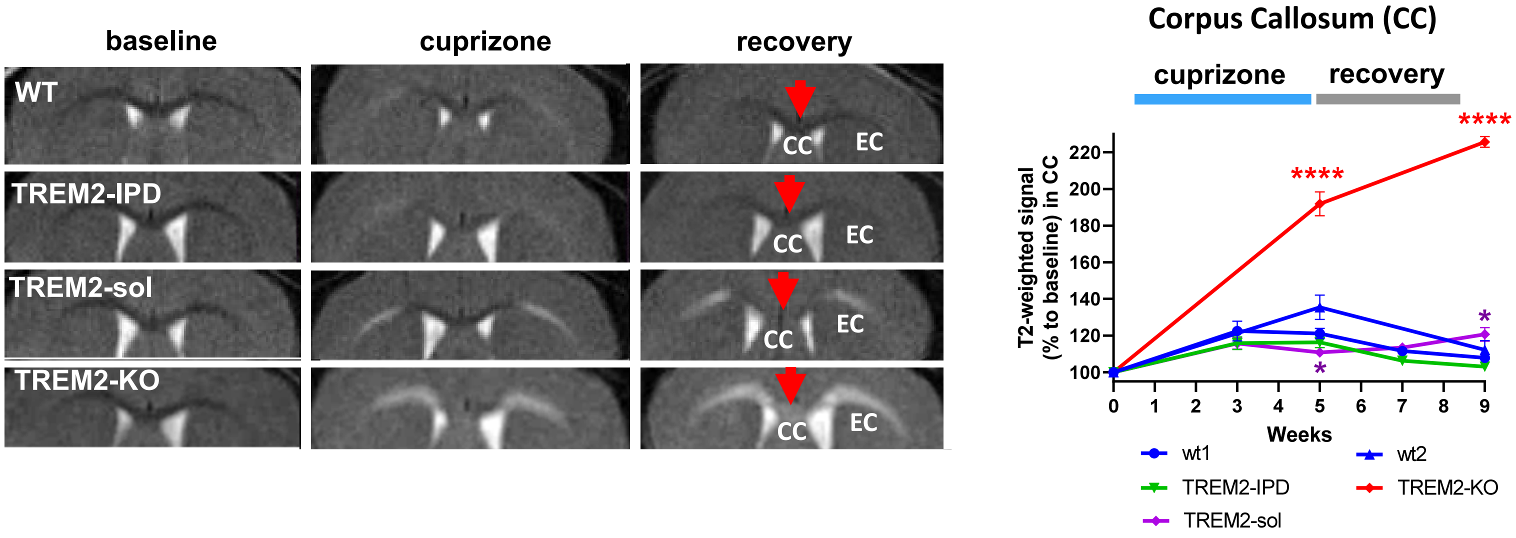

baseline
cuprizone
recovery
WT
TREM2-IPD
TREM2-sol
TREM2-KO
CC
EC
Corpus Callosum (CC)
cuprizone
recovery
T2-weighted signal (% to baseline) in CC
****
****
*
*
Weeks
wt1
wt2
TREM2-IPD
TREM2-KO
TREM2-sol

Figure S2

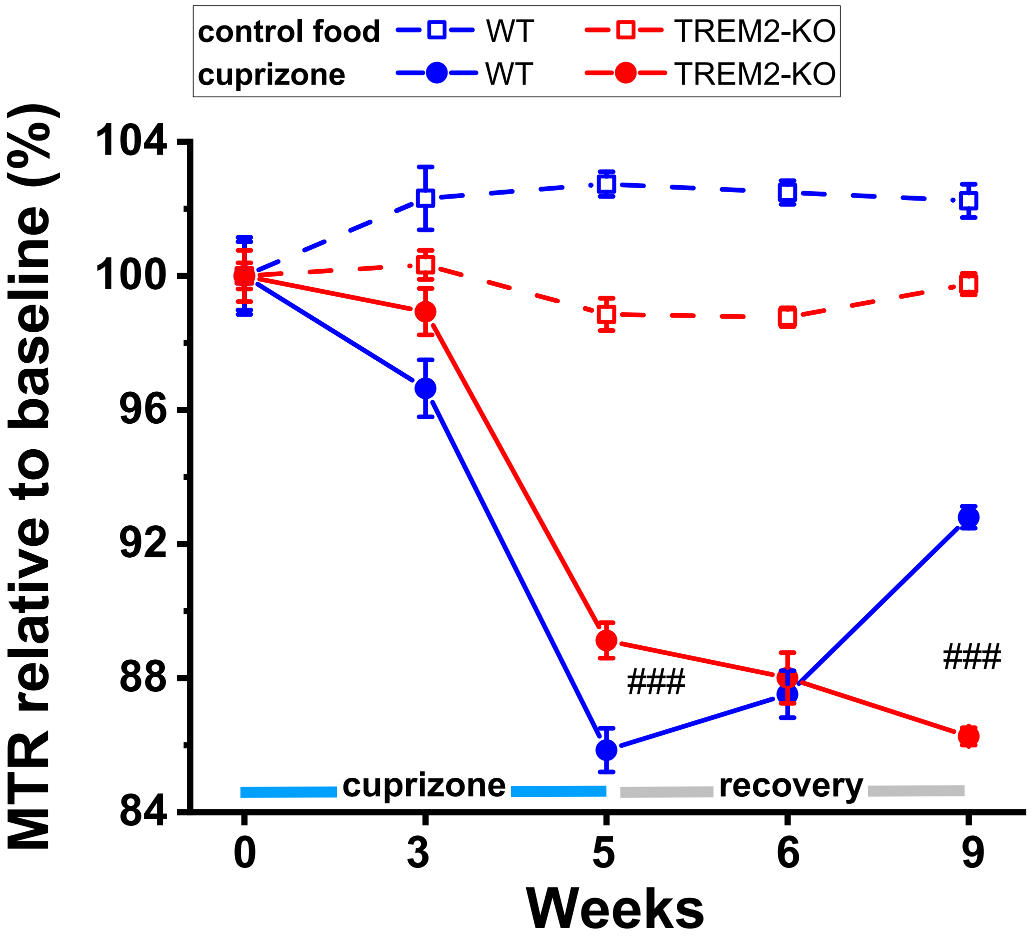
control food
WT
TREM2-KO
cuprizone
WT
TREM2-KO
MTR relative to baseline (%)
104
100
96
92
88
84
###
###
cuprizone
recovery
0
3
5
6
9
Weeks

Figure S3

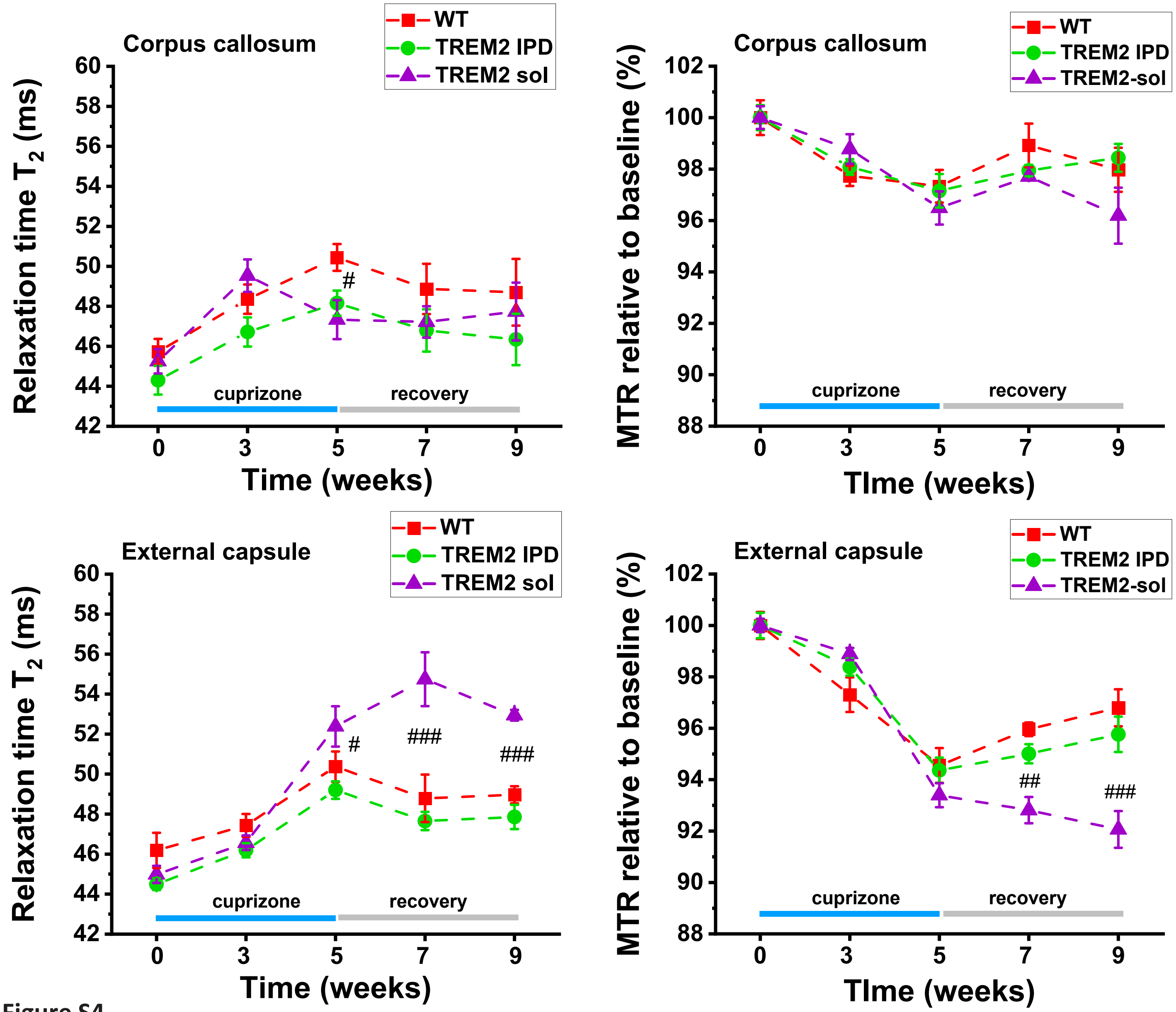
Corpus callosum
WT
TREM2 IPD
TREM2 sol
Relaxation time T_2 (ms)
cuprizone
recovery
#
Time (weeks)
Corpus callosum
WT
TREM2 IPD
TREM2-sol
MTR relative to baseline (%)
cuprizone
recovery
TIme (weeks)
External capsule
WT
TREM2 IPD
TREM2 sol
Relaxation time T_2 (ms)
#
###
###
cuprizone
recovery
Time (weeks)
External capsule
WT
TREM2 IPD
TREM2-sol
MTR relative to baseline (%)
##
###
cuprizone
recovery
TIme (weeks)

Figure S4

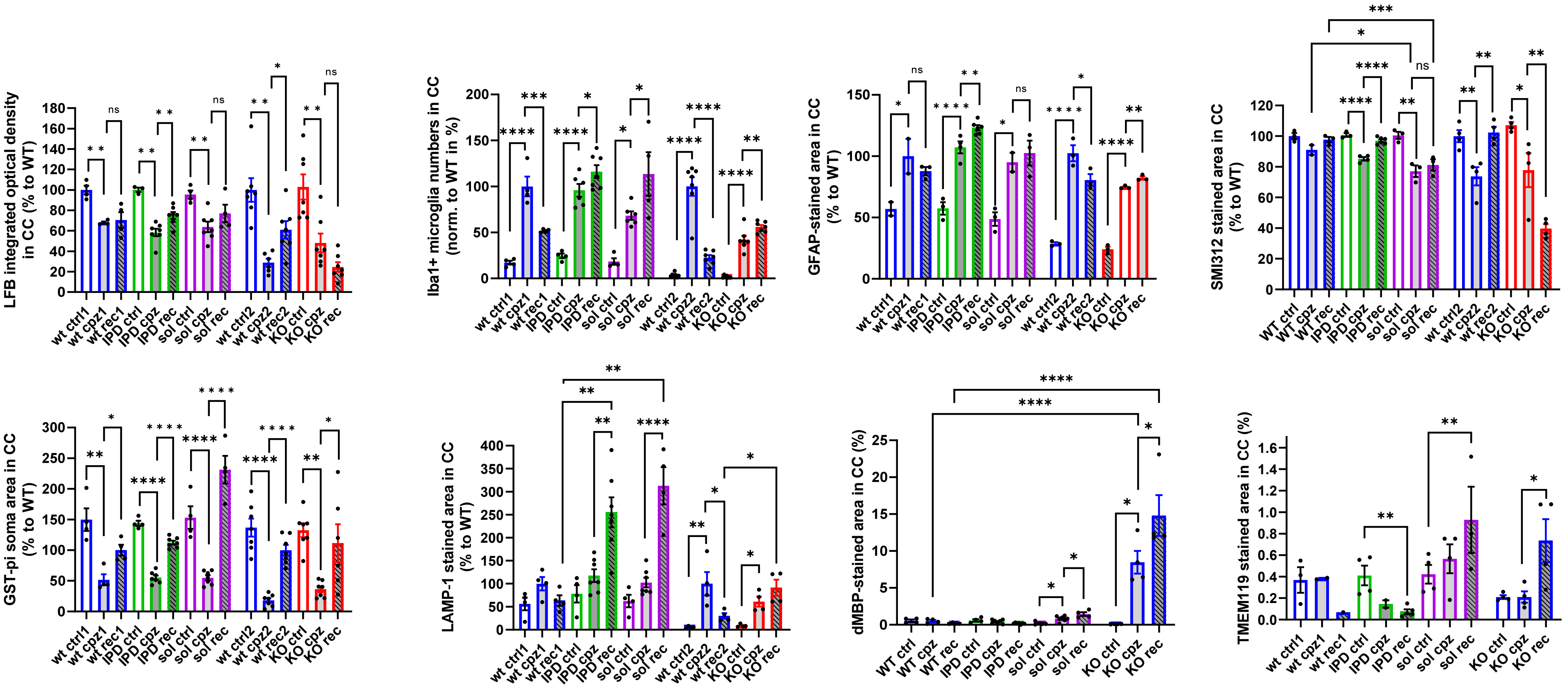
LFB integrated optical density in CC (% to WT)
Iba1+ microglia numbers in CC (norm. to WT in %)
GFAP-stained area in CC (% to WT)
SMI312 stained area in CC (% to WT)
GST-pi soma area in CC (% to WT)
LAMP-1 stained area in CC (% to WT)
dMBP-stained area in CC (%)
TMEM119 stained area in CC (%)
wt ctrl1
wt cpz1
wt rec1
IPD ctrl
IPD cpz
IPD rec
sol ctrl
sol cpz
sol rec
wt ctrl2
wt cpz2
wt rec2
KO ctrl
KO cpz
KO rec
WT ctrl
WT cpz
WT rec

Figure S5

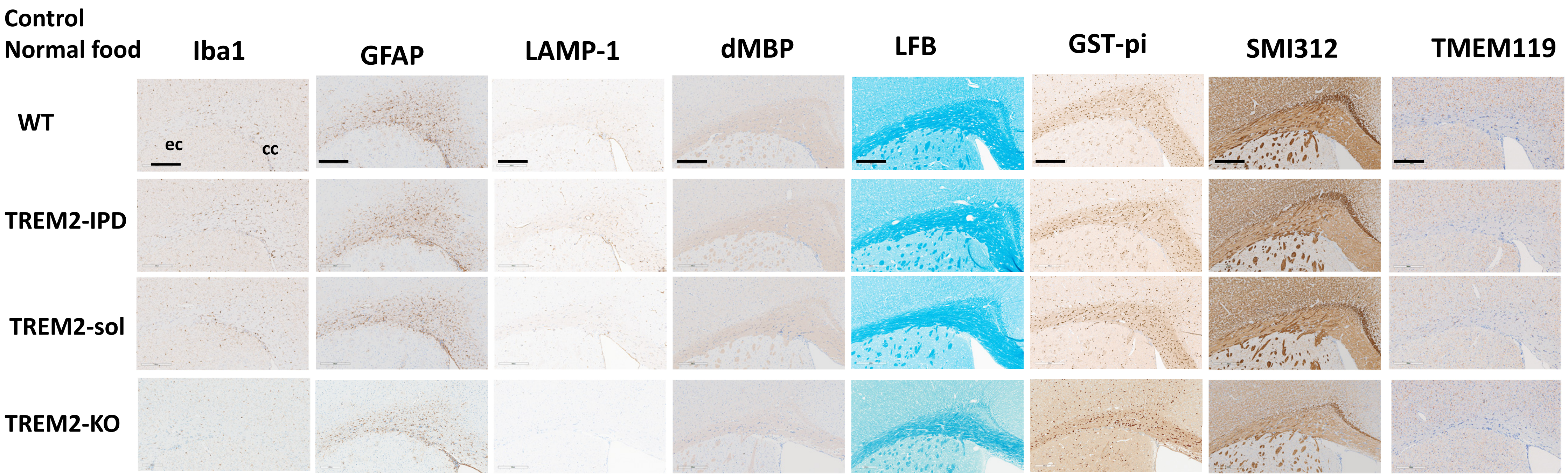
Control
Normal food
Iba1
GFAP
LAMP-1
dMBP
LFB
GST-pi
SMI312
TMEM119
WT
ec
cc
TREM2-IPD
TREM2-sol
TREM2-KO

Figure S6

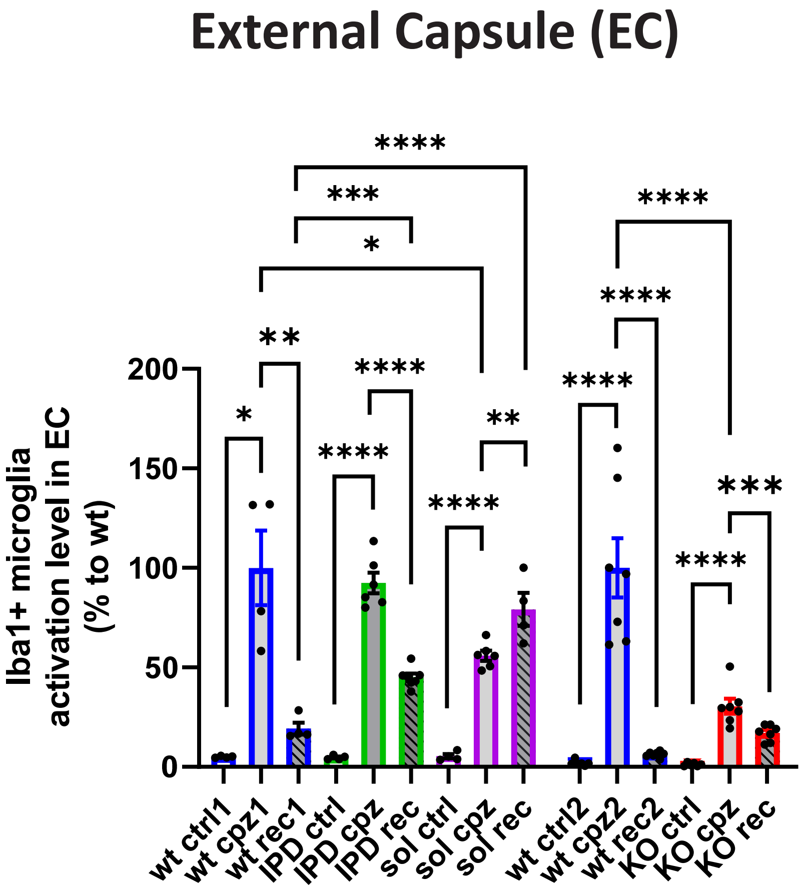
External Capsule (EC)
Iba1+ microglia activation level in EC (% to wt)
0
50
100
150
200
wt ctrl1
wt cpz1
wt rec1
IPD ctrl
IPD cpz
IPD rec
sol ctrl
sol cpz
sol rec
wt ctrl2
wt cpz2
wt rec2
KO ctrl
KO cpz
KO rec
*
**
****
****
****
**
*
***
****
****
****
****
****
***

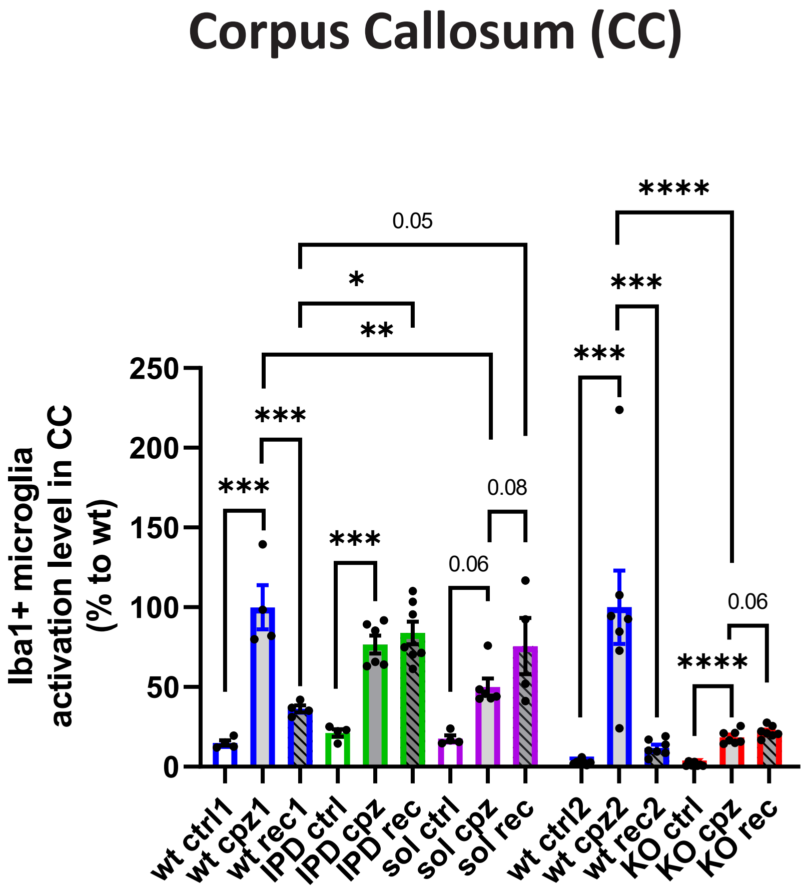
Corpus Callosum (CC)
Iba1+ microglia activation level in CC (% to wt)
0
50
100
150
200
250
wt ctrl1
wt cpz1
wt rec1
IPD ctrl
IPD cpz
IPD rec
sol ctrl
sol cpz
sol rec
wt ctrl2
wt cpz2
wt rec2
KO ctrl
KO cpz
KO rec
***
***
***
0.06
0.08
**
*
0.05
***
***
****
****
0.06

Figure S7

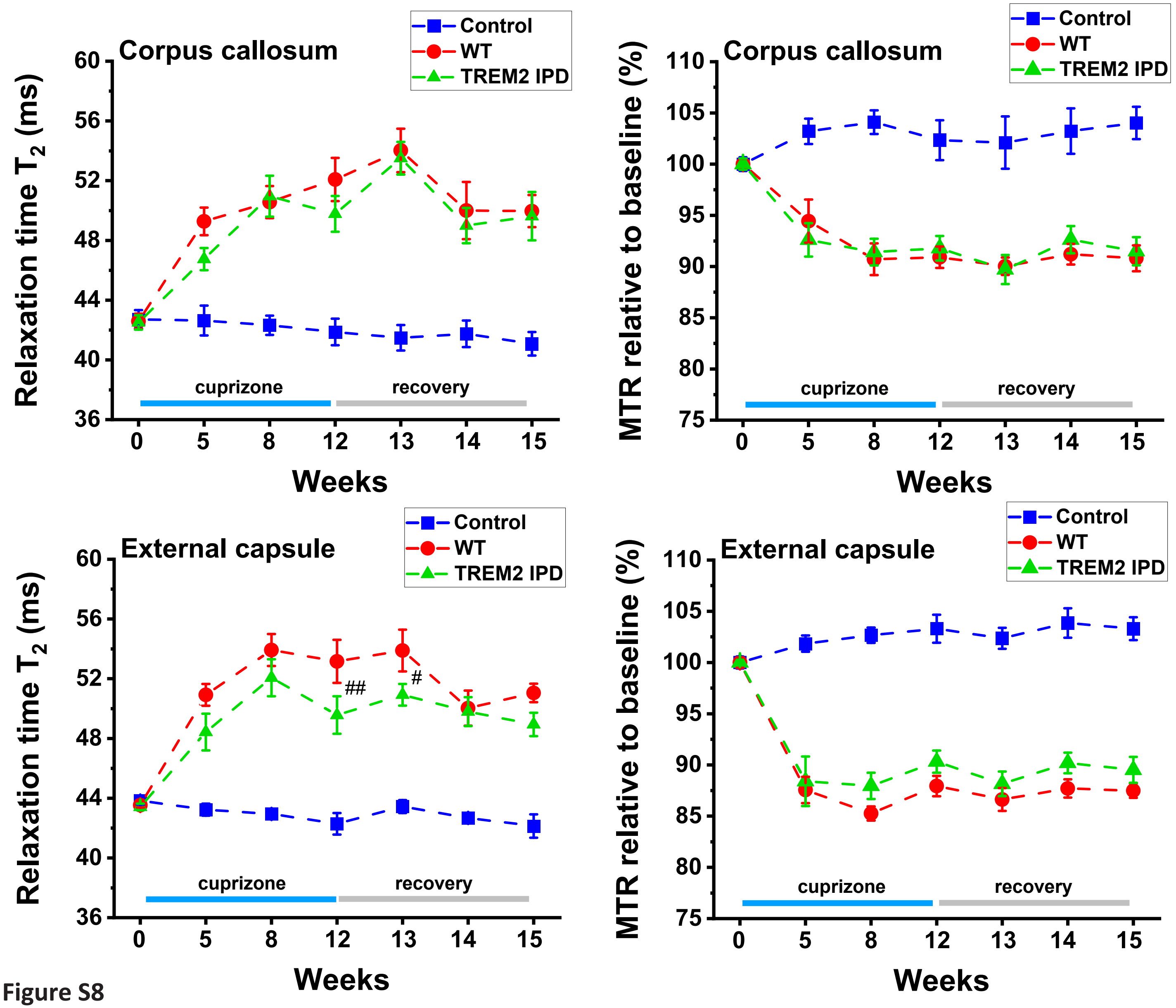

Corpus callosum
Relaxation time T_2 (ms)
Weeks
MTR relative to baseline (%)
External capsule
Control
WT
TREM2 IPD
cuprizone
recovery
##
#

Figure S8

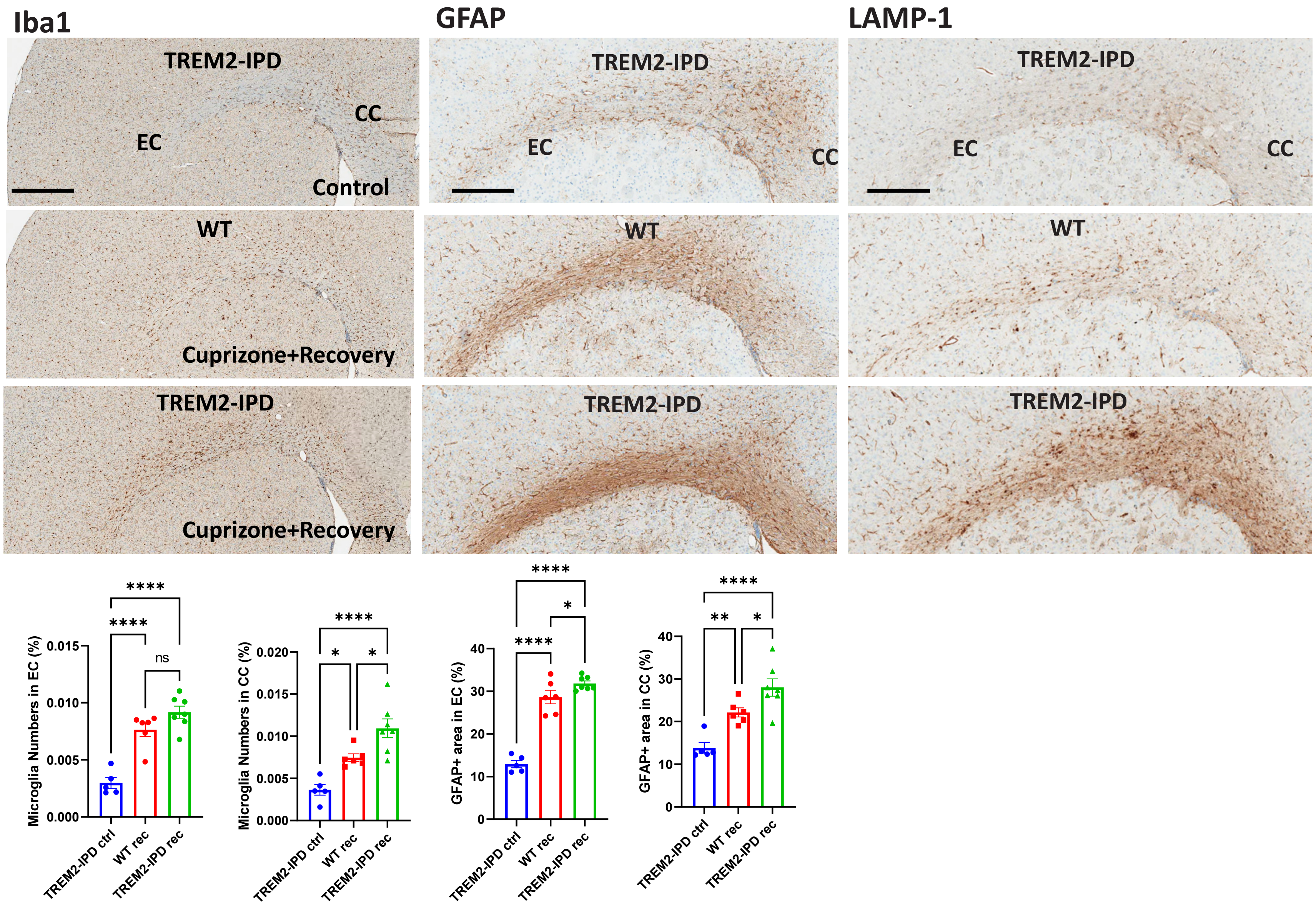
Iba1
GFAP
LAMP-1
TREM2-IPD
CC
EC
Control
WT
Cuprizone+Recovery
TREM2-IPD
Cuprizone+Recovery
Microglia Numbers in EC (%)
Microglia Numbers in CC (%)
GFAP+ area in EC (%)
GFAP+ area in CC (%)
TREM2-IPD ctrl
WT rec
TREM2-IPD rec
****
ns
*
**

Figure S9

Additional figure legends

Figure S1. Characterization of TREM2-sol in bone-marrow derived macrophages, brain and primary microglia. a Flow cytometry analysis of murine intracellular TREM2 in BMDM. MFI: median fluorescence intensity. Cells were either untreated or treated with the sheddase inhibitor DCP333 (5µM overnight, DPC) or the sheddase activator PMA (50ng/ml for 30min). The gate for TREM2 positive cells was set based on the isotype control (threshold 0.2% positive cells in the isotype control). The dotted line marks the background signal which is observed in TREM2-KO. **b** Mouse Trem2 qRT-PCR of total RNA from WT and TREM2-sol brains (age 12 months old). Normalized to Gapdh, 2^-ΔΔCt= 2^-(ΔCtTrem2-AvgΔCtControl/). n=6 per group. Statistics: Unpaired t-test two-tailed. **: p<0.01. **c** MIP1-β analysis in supernatants from BMDM for each genotype data in triplicates (n=3) at days in vitro (DIV) 7. **d** *In vitro* phagocytosis of primary microglia over 24h with 10µg pHrodo-myelin per well. The WT AUC of the integrated fluorescence intensities was set to 100%. All genotypes were compared to the other three, and all significant differences are displayed. In both experiments, data for each genotype data was generated in triplicates (n=3). Fluorescence measurements in wells without prey were used as controls (data not shown). Statistics: One-way ANOVA test with Tukey`s multiple comparisons test (***: p <.001, ****: p <0.0001).

Figure S2. T_2-weighted MRI signal analysis in the CC region for the 5-week cuprizone model in WT, TREK2-KO, TREM2-sol and TREM2-IPD mice. Groups consisted of mice treated for 5 weeks with control food (normal food) or 0.2% cuprizone in food and then switched back to control food (normal food) for the 4-week recovery. MRI measurements were performed at week 0 (baseline), week 3 and week 5 of cuprizone intoxication, at week 7 (2 weeks of recovery on control food) and at week 9 (4 weeks of recovery on control food). Mice were culled at week 9 immediately after the last MRI measurement. EC: external capsule, CC: corpus callosum. Group sizes: n=7-9 for all genotypes and at all time-point. Statistics: ANOVA with random effects comparisons indicated significant differences with respect to WT mice: *: p<0.05, ****: p<0.0001. For each group examined, T_2-weighted signals were significantly increased with respect to baseline values (significances not shown). Data are shown as means±SEM. wt1 is the respective wildtype group for the study with TREM2-IPD and TREM2-sol, wt2 is the wildtype group for the TREM2-KO study.

Figure S3. MTR analyses in the combined external capsule (EC) and corpus callosum (CC) region for the 5-week cuprizone and 4-week recovery model. Data (means±SEM) presented as relative to baseline values for each genotype. Group sizes: n=14 until week 5, n=7 until week 9 for all genotypes. Statistics: Anova with random effects. The levels of significance ###: p<0.001 correspond to comparisons betweenTREM2-KO and WT animals at the specified time points. WT: wildtype, TREM2-KO: TREM2 knockout

Figure S4. MRI analyses in external capsule (EC) and corpus callosum (CC) of TREM2-IPD and TREM2-sol mice for the 5-week cuprizone and 4-week recovery model. Data presented as means±SEM. Group sizes: n=7 for each genotype. Statistics: ANOVA with random effects. The levels of significance #: p<0.05, ##: p<0.01, ###: p<0.001 correspond to comparisons betweenTREM2-sol and WT animals at the specified time points. WT: wildtype, TREM2-IPD: TREM2 cleavage-reduced, TREM2-sol: TREM2 soluble-only.

Figure S5. Quantitative image analysis of histological stainings in CC. Luxol fast blue (LFB) optical density (OD) analysis of LFB (normalized to WT at control food), mature oligodendrocytes (GST-π soma area in %), myelin basic protein debris (dMBP-stained area in %), neurofilament (SMI312-stained area in %). Iba1-positive soma numbers (normalized to WT at week 5 cuprizone), LAMP-1 (lysosomal-associated membrane protein 1)-stained area in %, TMEM119 (homeostatic marker, TMEM119-stained area in %), astrocytes (GFAP-stained area in %). Group sizes: n=7 for all genotypes and time-points. Data shown as means±SEM. WT: wildtype, TREM2-IPD: TREM2 cleavage-reduced, TREM2-sol: TREM2 soluble-only, TREM2-KO: TREM2 knockout. Ctrl: control food, cpz: cuprizone food for 5 weeks, rec: recovery on control food for 4 weeks. CC: corpus callosum. Scale bars: 300µm (overview), 50 µm (close-up). Statistics: Holm-Sidak`s multiple comparison test one-way ANOVA (*: p<0.05, **: p<0.01, ***: p<0.001, ****: p<0.0001). Comparisons not indicated are non-significant. Wt ctrl1, wt cpz1 and wt rec1 are the respective wildtype groups for the study with TREM2-IPD and TREM2-sol, wt ctrl2, wt cpz2 and wt rec2 are the wildtype groups for the TREM2-KO study. Only statistical analysis within a study was performed. The analyses of dMBP and TMEM119 for the respective wt groups for TREM2-KO were omitted as no obvious signals were observed.

Figure S6. Brain histological analysis for WT, TREM2-IPD, TREM2-sol and TREM2-KO mice receiving control normal food showed no difference between groups. Quantitative image analysis is shown in Fig. 3 and 5. Luxol Fast Blue (LFB), mature oligodendrocytes (GST-π), myelin basic protein debris (dMBP), neurofilament (SMI312), Iba1, LAMP-1 (lysosomal-associated membrane protein 1), TMEM119 (homeostatic marker), astrocytes (GFAP). WT: wildtype, TREM2-IPD: TREM2 cleavage-reduced, TREM2-sol: TREM2 soluble-only, TREM2-KO: TREM2 knockout. ec: external capsule, cc: corpus callosum. Scale bars: 300µm.

Figure S7. Image analysis of Iba1-positive microglia activation in the EC and CC (normalized to WT in %) from the staining shown in Fig. 5. Microglia activation is calculated as microglia and proximal processes area normalized to non soma-associated processes area. Data are shown as means±SEM. WT: wildtype, TREM2-IPD: TREM2 cleavage-reduced, TREM2-sol: TREM2 soluble-only, TREM2-KO: TREM2 knockout. Ctrl: control food, cpz: cuprizone food for 5 weeks, rec: recovery on control food for 4 weeks. ec: external capsule, cc: corpus callosum. Statistics: Holm-Sidak`s multiple comparison test one-way ANOVA (*: $p<0.05$, **: $p<0.01$, ***: $p<0.001$, ****: $p<0.0001$). Comparisons not indicated are non-significant. wt ctrl1, wt cpz1 and wt rec1 are the respective wildtype groups for the study with TREM2-IPD and TREM2-sol, wt ctrl2, wt cpz2 and wt rec2 are the wildtype groups for the TREM2-KO study. Only statistical analysis within a study was performed.

Figure S8. MRI analyses of TREM2-IPD mice in the chronic cuprizone model. Data presented as means±SEM. Group sizes: n=7 for each genotype. Statistics: ANOVA with random effects. The levels of significance #$0.01<p<0.05$, ##$0.001<p<0.01$ correspond to comparisons between TREM2-IPD and WT animals at the specified time points. WT: wildtype, TREM2-IPD: TREM2 cleavage-reduced. Control refers to TREM2-IPD mice receiving normal food throughout the study.

Figure S9. Microglia (Iba1), astrocytes (GFAP) and lysosomal marker LAMP-1 were quantified in WT and TREM2-IPD in the chronic cuprizone model (12-week cuprizone treatment and 3-week recovery). Group sizes: n=5-7. Data are shown as means±SEM. WT: wildtype, TREM2-IPD: TREM2 cleavage-reduced. Ctrl: control food, rec: recovery on control food for 3 weeks. EC: external capsule, CC: corpus callosum. Scale bars: 500µm. Statistics: Holm-Sidak`s multiple comparison test one-way ANOVA (*: $p<0.05$, **: $p<0.01$, ****: $p<0.0001$, n.s.: not significant).
